# CXXC finger protein 1 is critical for T-cell intrathymic development through regulating H3K4 trimethylation

**DOI:** 10.1038/ncomms11687

**Published:** 2016-05-23

**Authors:** Wenqiang Cao, Jing Guo, Xiaofeng Wen, Li Miao, Feng Lin, Guanxin Xu, Ruoyu Ma, Shengxia Yin, Zhaoyuan Hui, Tingting Chen, Shixin Guo, Wei Chen, Yingying Huang, Yizhi Liu, Jianli Wang, Lai Wei, Lie Wang

**Affiliations:** 1 Institute of Immunology, Zhejiang University School of Medicine, Hangzhou 310058, China; 2 State Key Laboratory of Ophthalmology, Zhongshan Ophthalmic Center, Sun Yat-sen University, Guangzhou 510060, China; 3 Department of Biostatistics, University of Pittsburgh, Pittsburgh, Pennsylvania 15261, USA; 4 Division of Pulmonary Medicine, Allergy and Immunology, Department of Pediatrics, Children's Hospital of Pittsburgh of University of Pittsburgh Medical Center, Pittsburgh, Pennsylvania 15224, USA; 5 Core Facilities, College of Medicine, Zhejiang University, Hangzhou 310058, China

## Abstract

T-cell development in the thymus is largely controlled by an epigenetic program, involving in both DNA methylation and histone modifications. Previous studies have identified Cxxc1 as a regulator of both cytosine methylation and histone 3 lysine 4 trimethylation (H3K4me3). However, it is unknown whether Cxxc1 plays a role in thymocyte development. Here we show that T-cell development in the thymus is severely impaired in *Cxxc1*-deficient mice. Furthermore, we identify genome-wide Cxxc1-binding sites and H3K4me3 modification sites in wild-type and *Cxxc1*-deficient thymocytes. Our results demonstrate that Cxxc1 directly controls the expression of key genes important for thymocyte survival such as RORγt and for T-cell receptor signalling including Zap70 and CD8, through maintaining the appropriate H3K4me3 on their promoters. Importantly, we show that RORγt, a direct target of Cxxc1, can rescue the survival defects in *Cxxc1*-deficient thymocytes. Our data strongly support a critical role of Cxxc1 in thymocyte development.

## Abstract (web summary)

T cell development has been a classical model for understanding cell fate regulation by epigenetics. Here the authors show that Cxxc1 controls thymocyte development mainly through regulating several key genes, such as *Rorc*, *Zap70* and *Cd8*, which requires its H3K4me3 but not DNA methylation function.

## Graphical abstract

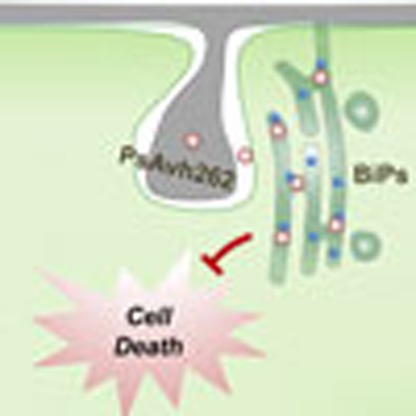

T-lymphocyte development in the thymus is tightly regulated by a network controlled by several key transcription factors^1^,^2^. Acquirement and expression of T-cell receptor (TCR) also play essential roles in generation of mature CD4^+^ or CD8^+^ T cells in the thymus. CD4 and CD8 double-negative T cells (DN) harbouring productive rearranged *Tcrb* genes start to express both CD4 and CD8 and progress to double-positive stage (DP), which composes the majority of thymocytes. DP thymocytes rearrange the *Tcra* locus and proceed to positive and negative selection based on their affinity to major histocompatibility complex (MHC) class I and MHC class II molecules. Thymocytes expressing mature αβTCRs and having appropriate binding affinity to MHC molecules can develop into CD4^+^CD8^−^ single-positive (CD4^+^ SP) helper or CD4^−^CD8^+^ single-positive (CD8^+^ SP) cytotoxic thymocytes^3^,^4^.

Recent studies clarified that epigenetic modifications such as DNA methylation and histone acetylations/methylations are crucial in controlling the commitment and maintenance of CD4 and CD8 lineages in the thymus. DNA methylation, which generally suppresses gene expression, establishes heritable epigenetic silencing of both CD4 and CD8 loci only in lineage-committed thymocytes^5^,^6^, while H3K(9,14)ac and H3K4me2, which often mark transcriptionally activated promoters or enhancers, promote stage-specific gene activation during DN–DP–SP step-wise development, with the most marked histone modification changes between DN and DP transition^7^.

In mammal cells, DNA methyltransferase 1 (DNMT1) maintains heritable DNA methylation patterns through every cellular DNA replication cycle. A huge catalogue of histone modifications in various organisms (including the lysine methylation/acetylation/ubiquitination/sumoylation, arginine methylation/citrulination and serine/threonine/tyrosine phosphorylation) are maintained by numerous epigenetic enzyme complexes. These cellular machineries responsible for epigenetic regulation also contribute to the regulation of thymocyte development. Histone deacetylases (HDACs) are required for positive and negative selection or survival of thymocytes^8^,^9^,^10^,^11^. Histone acetylation mediated by a subunit of the Hbo1 histone acetyltransferase complex Brd1 is also crucial in activating CD8 expression in thymocytes^12^. In addition, Manna *et al*.^13^ show that H3K27me3 demethylase Jmjd3 and Utx are required for CD4^+^ T-cell differentiation in the thymus through directly regulating the expression of sphingosine-phosphate receptor S1pr1. These studies suggest that epigenetic enzymes are important to coordinate thymocyte development. However, it is unclear how many of these epigenetic modifications direct the early T-cell differentiation.

CXXC finger protein 1 (Cxxc1) is defined as an unmethylated CpG-binding protein regulating DNA methylation through its DNA-binding domain. It is also a component of the H3K4 methyltransferase Setd1 complex and targets Setd1 and H3K4me3 to most CpG islands (CpGI) through its Setd1-interacting domain^14^,^15^,^16^,^17^,^18^. Deficiency of the *Cxxc1* in mice leads to peri-implantation lethality^19^. Its deletion in embryonic stem (ES) cells and human myeloid cell lines blocks their differentiation *in vitro*^14^,^20^. Moreover, its deletion in haematopoietic cells fails the haematopoiesis *in vivo*^21^. These data all suggest a pivotal role of Cxxc1 in development. Carlone *et al*.^14^ show that loss of Cxxc1 in ES cells reduces heterochromatin by decreasing DNMT1 activity and global cytosine methylation, in addition to inducing global H3K4me2 and H3K4me3 (ref. 15). In contrast, Clouaire *et al*. shows that Cxxc1 is required for H3K4me3 and deficiency of Cxxc1 in ES cells leads to marked loss of H3K4me3 at expressed CpGI-associated genes, with minimal consequences for transcription^22^. Furthermore, using chromatin immunoprecipitation sequencing (ChIP-seq) technology, Thomson *et al*.^18^ identify genome-wide Cxxc1-binding sites in a mixture of mouse brain tissues and find a notable concordance of Cxxc1 binding with H3K4me3 and non-methylated CpGI. Taken together, Cxxc1 may directly regulate gene expression through coordinating DNA methylation and H3K4me3 in an environment- and cell-type-specific manner.

To understand the role of Cxxc1 in intrathymic T-cell development, we generated Cxxc1 conditional knockout mouse strains and found that the intrathymic T-cell development was blocked severely in *Cxxc1*-deficient mice, due to impaired survival and TCR signalling in DP thymocytes. Using ChIP-seq technology, we identified genome-wide Cxxc1-binding sites and H3K4me3 modification changes in *Cxxc1*-deficient thymocytes. And we found that Cxxc1 directly regulated several key molecules in DP thymocytes, through introducing H3K4me3 modification on these genes' promoter regions. Moreover, Cxxc1's direct target RORγt could rescue the survival defects in *Cxxc1*-deficient thymocytes. Our data strongly support that Cxxc1-mediated epigenetic program is required for thymocyte development.

## Results

### Cxxc1 is required for T-cell development

Cxxc1 is a key epigenetic regulator and essential to embryogenesis. We also found that Cxxc1 was highly expressed in T-cell precursors in the thymus, especially at DN3 stage (Supplementary Fig. 1a,b). Therefore, we hypothesize that it may also be important for the development of T-cell thymic development. Since the *Cxxc1*-null mouse is embryonic lethal^23^, we generated the conditional *Cxxc1* allele (*Cxxc1*^fl^), in which the exons 4 and 5 were flanked by *loxp* sites (Supplementary Fig. 2a). Intercrossing between *Cxxc1*^+/−^ mice (generated by *Cxxc1*^T^ crossing with *PGK*^Cre^, see Methods) did not generate the *Cxxc1* deleted offspring, which verified the lethality of *Cxxc1*-null mouse (Supplementary Fig. 2b). To understand its role in thymocyte development, the *Cxxc1*^fl/fl^ mice (wild-type (WT) mice) were crossed with *hCD2*^Cre^ mice, leading to the deletion of functional CXXC1 protein in early stage of developing thymocytes^24^, as well as other immune cells. To tract *Cxxc1* deletion during T-cell development in the thymus, genomic DNA from sorted DN1, DN2, DN3, DN4 and DP subpopulations in WT and *Cxxc1*-deficient mice was tested for the expression of floxed and deleted *Cxxc1* alleles. We found that the deleted band appeared at DN1 and the floxed band disappeared at DP stage (Supplementary Fig. 2c). Consistent with the deletion of DNA, the *Cxxc1* messenger RNA (mRNA) was detectable in DN3 cells at the level of ∼20% of its control, and was almost undetectable in DP cells in *Cxxc1*-deficient mice (Supplementary Fig. 2d). The deletion of CXXC1 protein in DP cells was further confirmed by western blot (Supplementary Fig. 2e). Taken together, these data illustrate that the complete deletion of *Cxxc1* occurred at DP stage in our *Cxxc1*-deficient mice.

**Figure 1:**
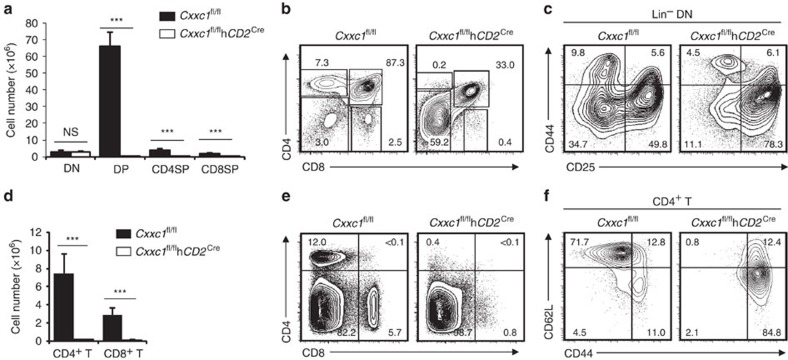
Intrathymic T-cell development was severely blocked in *Cxxc1*-deficient mice. (**a**) Quantification of DN, DP, CD4^+^ and CD8^+^ SP thymocyte subpopulations. The statistical significance was calculated by unpaired *t*-test (two tailed). ***P<0.001. NS, not significant. Error bars indicate s.d. (**b**) Flow cytometry analysis of the thymocytes from indicated mice using anti-CD4 and anti-CD8 antibodies. (**c**) Lineage-negative cells were analysed for profile of DN1–DN4 subsets: DN1 (CD44^+^CD25^−^), DN2 (CD44^+^CD25^+^), DN3 (CD44^−^CD25^+^) and DN4 (CD44^−^CD25^−^). (**d**) Quantitation of the absolute cell numbers of CD4^+^ and CD8^+^ T-cell subsets, as the product of total splenocytes multiplied by the percentage of cells found in that population. (**e**,**f**) Total splenocytes were analysed for CD4, CD8, CD44 and CD62L expression by flow cytometry. The statistical significance was calculated by unpaired *t*-test (two tailed). ***P<0.001. Error bars indicate s.d. Data are from six-mice analysis (**a**–**f**).

The size of thymus in *Cxxc1*-deficient mice was significantly reduced as compared with WT mice. Consistently, the thymic cellularity in *Cxxc1*-deficient mice was as low as ∼1% of its counterpart in WT mice, although the total number of DN cells was not significantly changed (Fig. 1a). The majority of thymocyte loss came from the drastic decrease in the numbers of DP, CD4 SP and CD8 SP thymocytes (Fig. 1a). In fact, most of thymocytes in *Cxxc1*-deficient mice were either DP (33%) or DN (59.2%) cells, while both CD4 and CD8 SP cells were almost completely abolished (Fig. 1b). Further analysis of the DN subpopulations showed an increase of CD25^+^ DN3 cells and a decrease of DN4 cells in *Cxxc1*-deficient mice comparing with the WT mice (Fig. 1c), despite an incomplete deletion of *Cxxc1* at DN3 stage (Supplementary Fig. 2c,d), suggesting that the transition from DN3 to DN4 might be compromised due to partial reduction of Cxxc1 expression. Subsequently, the total number and composition of peripheral T cells were also remarkably changed in *Cxxc1*-deficient mice. CD4^+^ and CD8^+^ splenocytes were mostly depleted (Fig. 1d,e) and the a few survived CD4^+^ cells acquired effector/memory phenotype with elevated expression of CD44 and decline expression of CD62L (Fig. 1f), reflecting the developmental defect caused by *Cxxc1* deficiency in the thymus.

To further confirm the function of Cxxc1 in thymocyte development, we crossed the *Cxxc1*^fl/fl^ with Lck^Cre^ mice, in which the deletion of *Cxxc1* started at DN3 stage. Lck^Cre^-mediated deletion of *Cxxc1* led to the similar thymocyte and peripheral T cell developing defects as seen in *Cxxc1*^fl/fl^h*CD2*^Cre^ mice (Supplementary Fig. 3a–e), although less severe defects were found in *Cxxc1*^fl/fl^*Lck*^Cre^ due to the late expression of Cre recombinase and possible leaky deletion of the *Cxxc1* gene by *Lck*^Cre^ mice^25^. On the other hand, existence of the substantial CD4^+^ and CD8^+^ SP populations in *Cxxc1*^fl/fl^*Lck*^Cre^ might also be due to an incomplete deletion of *Cxxc1* gene (Supplementary Fig. 3f,g). Furthermore, we also used the *Cxxc1*^fl/fl^CreERT2^+^ and OP9-DL1 cell system to test the transition of DN to DP stage *in vitro*^26^. The DN3 cells were sorted from *Cxxc1*^fl/fl^CreERT2^+^ mice and co-cultured with OP9-DL1 cells in presence or absence of 4-hydroxytamoxifen (4-HT; which initiated the access of Cre recombinase in nucleus and led to the deletion of *Cxxc1 in vitro*, shown in Supplementary Fig. 3h,i) for 5 days. As shown in Supplementary Fig. 3j,k, *Cxxc1* deletion *in vitro* also resulted in the reduction of DN–DP transition and depletion of SP cells. Taken together, our data indicate that Cxxc1 is required for T-cell development. Given that the complete deletion of *Cxxc1* occurred at DP stage and a severe disruption of DP thymocyte development was found in the *Cxxc1*-deficient mice, we therefore focused our further study on DP thymocytes.

### Cxxc1 regulates T-cell development with its H3K4me3 function

Previous reports have demonstrated that Cxxc1 is a component of Setd1 complex. We also found that Cxxc1 indeed interacted with SETD1 in DP thymocytes (Supplementary Fig. 4a). Cxxc1 interacts with unmethylated CpG DNA through its CXXC domain (N terminal). Three regions of Cxxc1 between amino acid 169 and 493 are found to mediate its interaction with DNMT1 and stabilize DNMT1 protein for regulating DNA methylation^27^. A point mutation (C169A) interrupting DNA-binding activity of Cxxc1 ablates its DNA-binding ability in ES cells^17^. On the other hand, Cxxc1 interacts with Setd1 H3K4 methyltransferase complex through SID domain (C terminal) to regulate histone methylation. A point mutation (C375A) interrupting the Setd1-interacting activity of Cxxc1 ablates its function in H3K4 methylation regulation in ES cells, but not its interaction with DNMT1 (refs 17, 27). Using the *in vitro* T-cell development system and different vectors expressing mutated Cxxc1 proteins in *Cxxc1*-deficient thymocytes, we tried to test that functional domains within Cxxc1 were necessary for its role in T-cell development. We first generated vectors expressing the full-length *Cxxc1* gene (Cxxc1-IRSE-GFP, with *Cxxc1* 1–656 aa), N-terminal fragment of *Cxxc1* (1-367-IRES-GFP) that has DNA-binding activity, C-terminal fragment of *Cxxc1* (361-656-IRES-GFP) that interacts with the Setd1 complex, full-length *Cxxc1* with the point mutation C169A (C169A-IRES-GFP) in which the DNA-binding activity was abolished and full-length *Cxxc1* with the point mutation C375A (C375A-IRES-GFP) in which the Setd1-interacting activity was abolished, respectively (Supplementary Fig. 4b). Importantly, expression of Cxxc1 by the two vectors with point mutations was comparable to the one without point mutation in DP thymocytes (Supplementary Fig. 4c), which was consistent with the previous report in ES cells^17^.

**Figure 2:**
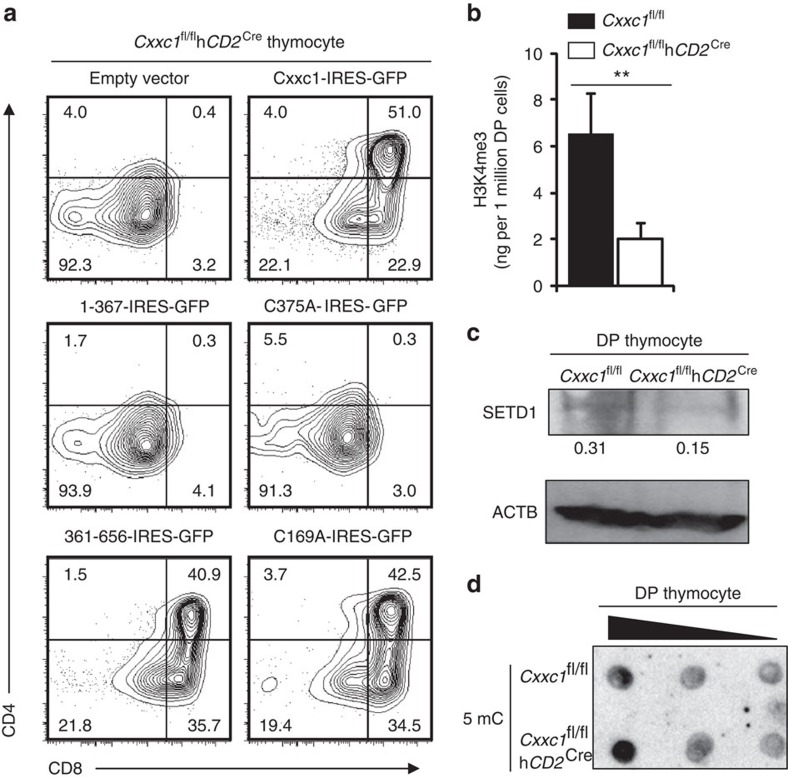
Setd1-interacting domain of Cxxc1 is required for intrathymic T-cell development. (**a**) DN3 cells from *Cxxc1*-deficient mice were sorted and transfected with indicated retrovirus. GFP^+^ thymocytes were analysed by fluorescence-activated cell sorting after co-culturing with OP9-DL1 for 5 days. Data comes from four independently sorted DN3 samples (each from three mice). (**b**) Detection of total H3K4me3. Crude histone protein were extracted from DP thymocytes sorted from control or *Cxxc1*-deficient mice using acid extraction, the protein was subjected to H3K4me3 ELISA analysis. Data comes from three separately sorted DP cell sets (each from two mice). The statistical significance was calculated by unpaired *t*-test (two tailed). **P<0.01. Error bars indicate s.d. (**c**) Detection of Setd1 protein level. DP thymocytes from *Cxxc1*-deficient and control mice were used for western blot analysis. Number under lanes indicate densitometry of Setd1 (relative to Actb at same lane, below). Data comes from three separately sorted DP cell sets (each from three mice). (**d**) Dot blotting showed total 5 mC levels in sequentially diluted genomic DNA from indicated DP thymocytes. Data are representative of three separate experiments (each from two mice).

Ectopic expression of full-length *Cxxc1* in WT DN cells had trivial effects on thymocyte development (Supplementary Fig. 4d), but it could fully rescue the T-cell developmental defects in *Cxxc1*-deficient cells (Fig. 2a). Intriguingly, the C-terminal fragment of *Cxxc1* gene (361-656-IRES-GFP) rather than the N-terminal fragment of *Cxxc1* gene (1-367-IRES-GFP) was able to rescue T-cell development in *Cxxc1*-deficient thymocytes (Fig. 2a). Similarly, the C375A muted full-length protein (C375A-IRES-GFP) with defect in interacting with Setd1 was unable to rescue impaired T-cell development in *Cxxc1*-deficient thymocytes, while the C169A muted full-length protein (C169A-IRES-GFP) was still able to convert DN to DP cells (Fig. 2a). These data strongly indicated that the Setd1 interacting, but not the DNA-binding domain in *Cxxc1* is crucial for thymocyte development.

Cxxc1 forms a complex with Setd1 to promote H3K4 methylation and transcription activation. Therefore, we next examined whether H3K4 trimethylation was affected in *Cxxc1*-deficient thymus. The crude histone extracted from WT or *Cxxc1*-deficient DP thymocytes were subjected to detection of total H3K4me3 levels by enzyme-linked immunosorbent assay (ELISA). As shown in Fig. 2b,c, the total H3K4me3 level and SETD1 expression in *Cxxc1*-deficient cells were remarkably lower than that in WT DP thymocytes, suggesting an important role of Cxxc1 in maintaining the H3K4me3 level in DP thymocytes. However, we did not find any changes in overall DNA methylation (Fig. 2d) or DNMT1 protein levels (Supplementary Fig. 4e) in *Cxxc1*-deficient DP thymocytes. These data demonstrated that Cxxc1 may function through regulating H3K4me3 rather than DNA methylation in DP thymocytes.

### Cxxc1 binding and H3K4me3 modification profiles in DP cells

**Figure 3:**
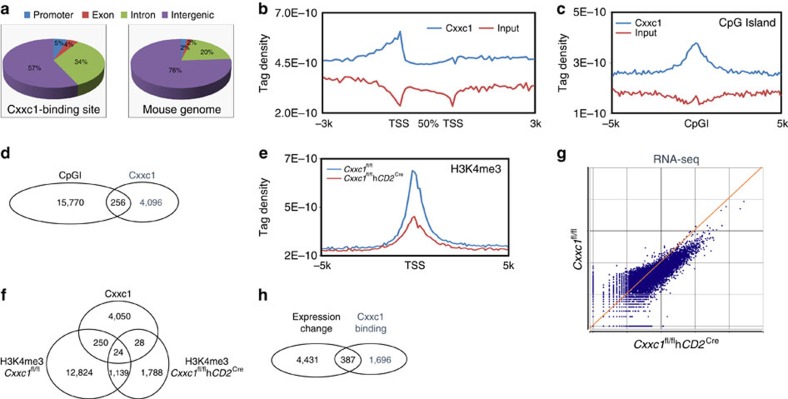
Genome-wide maps of Cxxc1 binding and H3K4me3 modification in DP thymocytes. (**a**) Distribution of Cxxc1-binding peaks in DP thymocytes, and distribution of the genetic features across the whole-mouse genome (mm9). (**b**) Distribution of Cxxc1-binding peaks across extended gene bodies. The tag density of Cxxc1 binding was calculated on gene bodies (between transcription start site, TSS and transcription termination site, TTS), as well as 3-kb upstream of TSS and 3-kb downstream of TTS regions of all RefSeq (mm9) genes. Uniquely, mapped tags were summarized in 100-bp windows for promoter regions and in 5% of gene body sequences. All window tag counts were normalized by the total number of bases in the windows and the total read number of the given sample. (**c**) Enrichment of Cxxc1-binding peaks on CpG islands (CpGI). The tag density of Cxxc1 binding was calculated on CpG islands and 5-kb flanking regions. (**d**) The number of genomic regions where CpGI and Cxxc1-binding sites were colocalized. (**e**) Distribution of H3K4me3 modification around 5-kb regions flanking TSS of all RefSeq (mm9) genes in WT and *Cxxc1*-deficient DP thymocytes. (**f**) Overlapped regions between Cxxc1-binding sites and H3K4me3 sites in WT and *Cxxc1*-deficient DP thymocytes. (**g**) Scatter plot of expression of all genes in WT and *Cxxc1*-deficient DP thymocytes, detected by RNA-seq analysis. (**h**) The number of genes with both Cxxc1-binding sites on their extended gene bodies (10-kb upstream of TSS plus gene body) and gene expression changes (over twofolds) between WT and *Cxxc1*-deficient DP thymocytes.

To fully understand the molecular mechanism by which Cxxc1 regulates thymocyte development, we carried out ChIP-seq analysis to map genome-wide Cxxc1-binding sites in WT DP cells, as well as in *Cxxc1*-deficient DP cells serving as a negative control. Using the peak calling program SICER V1.1 (false discovery rate (FDR)=10^−5^, *Cxxc1*-deficient sample and input as negative controls), we identified 4,352 genomic regions that were directly bound by Cxxc1 in WT DP thymocytes (Supplementary Table 1). Among these Cxxc1-associated sites, ∼43% of them located within extended gene bodies (5% promoter, 4% exon and 34% intron; Fig. 3a; Supplementary Table 1). This represented a low degree of enrichment at promoter–gene body regions as compared with the distribution of these genomic features across the mouse genome (Fig. 3a). The average binding location analysis also indicated that Cxxc1-binding activity was accumulated at transcription start sites (TSS) (Fig. 3b). Importantly, a unique feature for Cxxc1 binding was the relative high-binding activity between TSS and ∼2 kb promoter regions, suggesting that some Cxxc1-associated transactivation complexes may also bind proximal promoters in addition to TSS. Moreover, the motif analysis of Cxxc1-binding sites also identified multiple transcription factors, including TCF3, RUNX1, BCL6B, RARG and RARA as potential co-binding partners, which potentially coordinated the transcriptional regulation of Cxxc1 targets (Supplementary Fig.5a).

We found that Cxxc1-dependent DNA methylation regulation might be dispensable during thymocyte differentiation (Fig. 2a,d). We here examined again whether Cxxc1-binding sites were associated with CpGI in DP thymocytes. As shown in Fig. 3c, Cxxc1 binding was enriched at the centre of CpGI. However, only 6% of Cxxc1-binding sites were found colocalized with CpGI in DP cells (Fig. 3d). This is significantly different with previous finding that 93% of Cxxc1-binding sites overlap with CpGI in brain tissues (Thomson *et al*.^18^). In fact, only 13% of Cxxc1-binding sites found in DP thymocytes were also found in brain tissues (Supplementary Fig. 5b), indicating a tissue-specific binding profile of Cxxc1 may decide its primary function in a cell-type-dependent manner.

Our results above showed that Cxxc1 was crucial for the maintenance of overall H3K4me3 levels in DP thymocytes. We therefore carried out ChIP-seq analysis to map the genome-wide H3K4me3 modifications in DP cells. Interestingly, a significant reduction in the number of H3K4me3 peaks was found in *Cxxc1*-deficient DP cells (Fig. 3e; Supplementary Table 1). However, only very few Cxxc1-binding sites were found colocalized with H3K4me3 marks (Fig. 3f).

Published work reported a minimal correlation between H3K4me3 reduction and downregulation of gene expression in ES cells (Clouaire *et al*.^22^). We then asked whether in DP thymocytes a Cxxc1-dependent H3K4me3 reduction would lead to gene expression changes. RNA sequencing (RNA-seq) analysis of WT and *Cxxc1*-deficient DP thymocytes clearly demonstrated that the majority of genes showed reductions in their expression in *Cxxc1*-deficient DP thymocytes (Fig. 3g). We identified 2,083 genes at which loci the Cxxc1 directly bound within extended gene bodies (up to −10 kb from TSS). Among these genes, 387 genes had at least twofold expression changes in *Cxxc1*-deficient DP cells as compared with WT cells (Fig. 3h; Supplementary Dataset 1), and the expression of 85% of them was downregulated due to the deficiency of *Cxxc1* and the reduction of H3K4me3 on their promoters (Supplementary Fig. 5c). A gene ontology analysis of these 387 genes using DAVID^28^ revealed that the genes responsible for chromatin modification and regulation of thymic T-cell selection and differentiation, such as *Cd8a*, *Lck*, *Zap70* and *Rorc*, were the major targets of Cxxc1 in DP cells (Supplementary Table 2). These data indicate that Cxxc1 controls T-cell development through epigenetically regulating the expression of key genes involved in intrathymic T-cell differentiation.

### Cxxc1 epigenetically regulates key genes in T-cell development

**Figure 4:**
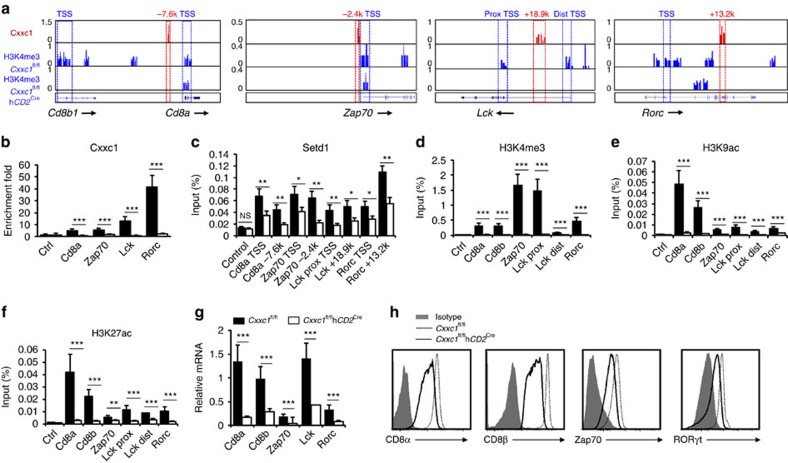
Epigenetic regulation of Cxxc1 target genes in DP thymocytes. (**a**) Genome browser views of murine *Cd8a*–*Cd8b*, *Zap70*, *Lck* and *Rorc* loci, with Cxxc1-binding signals (red) in WT DP thymocytes and H3K4me3 signals (blue) in WT and *Cxxc1*-deficient DP thymocytes. (**b**–**f**) Detection of Cxxc1 binding (**b**), Setd1 binding (**c**) and H3K4me3 (**d**), H3K9ac (**e**) and H3K27ac (**f**) modifications by ChIP–qPCR on *Cd8a*–*Cd8b*, *Zap70*, *Lck* and *Rorc* loci in WT and *Cxxc1*-deficient DP thymocytes. An intergenic region of chromatin 2 served as control. Data comes from three independently sorted sample sets (for H3K4me3, H3K9ac and H3K27ac, each from two mice; and for Cxxc1 and Setd1, each from four mice). The statistical significance was determined by unpaired *t*-test (two tailed). NS, not significant. **P<0.01, ***P<0.001. Error bars indicate s.d. (**g**) mRNA expression of *Cd8a*, *Cd8b*, *Zap70*, *Lck* and *Rorc* in WT and *Cxxc1*-deficient DP thymocytes, detected by quantitative real-time PCR. (**h**) Protein levels of CD8α, CD8β, ZAP70 and RORγt detected by fluorescence-activated cell sorting in control and *Cxxc1*-deficient DP thymocytes. Data are from three-separate mice experiments (**g**,**h**). The statistical significance was determined by unpaired *t*-test (two tailed). ***P<0.001 Error bars indicate s.d.

TCR signalling is vital for T-cell selection in the thymus and survival signal is also required for T cells to pass the positive and negative selection stage of DP cells to become mature SP T cells. ChIP-seq data showed that Cxxc1 bound to upstream or gene body enhancers of *Cd8a*, *Zap70*, *Lck* and *Rorc* gene loci, which was associated with a significant decrease in the H3K4me3 marks on these genes' promoter regions in *Cxxc1*-deficient DP cells (Fig. 4a). Direct binding of Cxxc1 in WT cells and reduction in H3K4me3 in *Cxxc1*-deficient cells on these gene loci were confirmed by ChIP–PCR assays (Fig. 4b,d). Importantly, we also found that the binding of SETD1 and CXXC1 were colocalized to the same promoter regions on *Cd8a*, *Zap70*, *Lck* and *Rorc* gene loci in WT DP thymocytes, which was significantly reduced in *Cxxc1*-deficient DP thymocytes (Fig. 4b,c). In addition, we found that both H3K9ac and H3K27ac (two histone modifications associated with activated chromosome), but not H3K27me3 and H3K9me2 (two histone modifications associated with inactivated chromosome) associated with these four gene loci (Fig. 4e,f; Supplementary Fig. 6a,b). Importantly, these active histone modifications on the four gene loci were all found lost in *Cxxc1*-deficient DP thymocytes (Fig. 4d–f), while on the loci that Cxxc1 did not appear to bind, such as *Bdnf* gene locus, these active histone modifications were unchanged (Supplementary Fig. 6c–f).

Our RNA-seq data showed that deletion of *Cxxc1* in DP cells led to 6.9-, 2.8-, 4.2- and 3.7-fold reduction in the expression of *Cd8a*, *Zap70*, *Lck* and *Rorc*, respectively (Supplementary Dataset 1). These expression changes were verified by real-time quantitative PCR (qPCR) assays on biological replicated samples (Fig. 4g). Importantly, we were able to detect significant reduction of CD8α, CD8β, ZAP70, and RORγt proteins in *Cxxc1*-deficient thymocytes (Fig. 4h). The protein level of lymphocyte protein tyrosine kinase (LCK) detected by western blot was not changed in spite of its RNA reduction in *Cxxc1*-deficient DP cells (Supplementary Fig. 6g). In addition, we have performed qPCR, methylated DNA immunoprecipitation (MeDIP)–qPCR and ChIP experiments across different thymocyte developmental stages to examine the gene expression, methylation status, H3K4me3 modification, as well as Cxxc1- and Setd1-binding status on the promoters of several target genes, respectively. We found a significant association between gradual acquisition of gene expression and reduction in DNA methylation, increase in H4K4me3 level, and binding of Cxxc1 and Setd1 on gene promoters (Supplementary Fig. 7a–e). These results suggested that Cxxc1-dependent epigenetic regulatory program may control genes important for DP survival and TCR signalling during thymocyte development.

### Cxxc1 is required for TCR signalling in DP thymocytes

**Figure 5:**
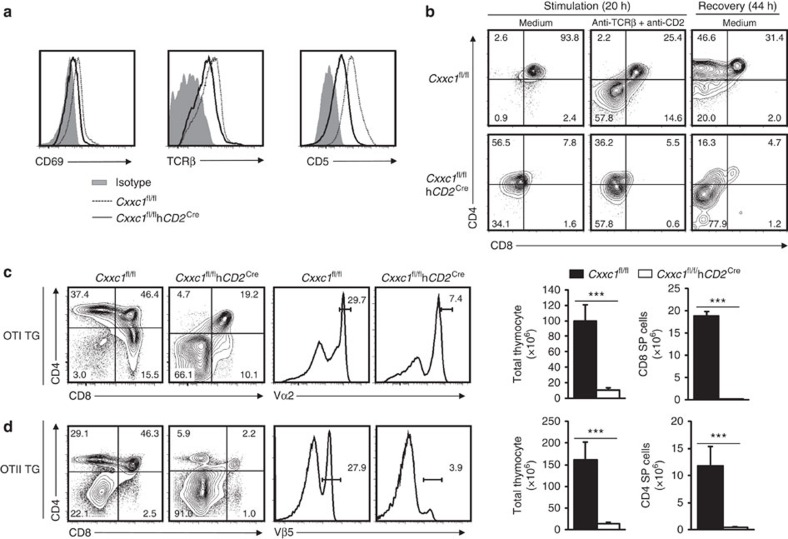
Cxxc1 was crucial for TCR signalling in DP thymocytes. (**a**) Flow cytometry analysis of CD69, CD5 and TCRβ in indicated DP thymocytes. Data are from three-mice analysis. (**b**) DP thymocytes sorted form indicated mice were cultured with medium alone (unstimulated; left), or with anti-TCRβ and anti-CD2 overnight and analysed immediately (middle), or washed and incubated for another 24 h in medium without antibodies (right). And analysis of live cells by flow cytometry. Data comes from three independently sorted DP cell sets (each from three mice). (**c**,**d**) Flow cytometry of thymocytes from control or *Cxxc1*-deficient mice bearing a transgene encoding the MHC class I-restricted OT-I TCR (**c**) or the MHC class II-restricted OT-II TCR (**d**). Left panel, staining of anti-CD4 and anti-CD8 on total thymocytes. Middle panel, staining with antibody to the OTI-specific variable region Vα5 (**c**) or the OTII-specific variable region Vβ5 (**d**). Right panel, quantitation of total thymocytes, CD8 SP thymocytes (**c**) or CD4 SP thymocytes (**d**). Data are from four-mice experiments. The statistical significance was determined by unpaired *t*-test (two tailed). ***P<0.001. Error bars indicate s.d.

The finding that Cxxc1 controls the expression of key players involved in TCR activation led us to hypothesize that the developmental defects found in *Cxxc1*-deficient thymocytes might be partially due to impaired TCR signalling. In addition to the reduction of CD8 and Zap70 expression, we found that the expression of other TCR activation markers such as CD69, TCRβ and CD5 was all significantly reduced in *Cxxc1*-deficient DP thymocytes (Fig. 5a). Furthermore, TCR activation-induced phosphorylation of both ERK and JNK proteins was also attenuated in *Cxxc1*-deficient DP thymocytes (Supplementary Fig. 8a). Taken together, our data demonstrated that Cxxc1 directly or indirectly controls the expression and activation of molecules involved in TCR signalling in DP thymocytes.

To investigate whether Cxxc1 regulates the positive selection of thymic T cells, we isolated DP thymocytes from WT- and *Cxxc1*-deficient mice, and stimulated them with anti-CD2 and anti-TCRβ for 20 h, followed by a recovery phase of 44 h. The WT DP thymocytes downregulated their CD4 and CD8 expression, and passed through CD4^+^CD8^in^ transitional stage (Fig. 5b)^29^,^30^,^31^. However, the *Cxxc1*-deficient DP thymocytes failed to generate the CD4^+^CD8^in^ population (Fig. 5b). Our results demonstrated that deficiency of *Cxxc1* led to defects in positive selection of thymic T cells.

The low expression of TCRβ in *Cxxc1*-deficient DP thymocytes raised a possibility that Cxxc1 might regulate TCR expression during T-cell development, which may account for the impaired TCR signalling. Hence, we crossed MHC class I-restricted or MHC class II-restricted TCR transgenic (TG) mice (OTI or OTII) with *Cxxc1*-deficient mice to test whether exogenously expressed TCR would rescue the TCR signalling defects found in *Cxxc1*-deficient mice. As shown in Fig. 5c,d, the frequency of DP and SP cells, as well as the total numbers of thymocytes and CD8 SP cells in OTI-TG-*Cxxc1*^fl/fl^ *hCD2*^cre^ or CD4 SP cells in OTII-TG-*Cxxc1*^fl/fl^ *hCD2*^cre^ mice were still remarkably lower than their WT counterparts; the frequency of Vα2^high^ thymocytes in OTI-TG-*Cxxc1*^fl/fl^ *hCD2*^cre^ or Vβ5^+^ thymocytes in OTII-TG-*Cxxc1*^fl/fl^ *hCD2*^cre^ mice was still significantly lower than their WT counterparts, indicating that productive TCR expression is not able to rescue the TCR signalling defects in *Cxxc1*-deficient mice. In summary, our results indicated that Cxxc1 is required for TCR signalling transduction through regulating many key molecules mediating TCR activation in DP thymocytes.

### Cxxc1 controls DP thymocytes' survival by regulating RORγt

**Figure 6:**
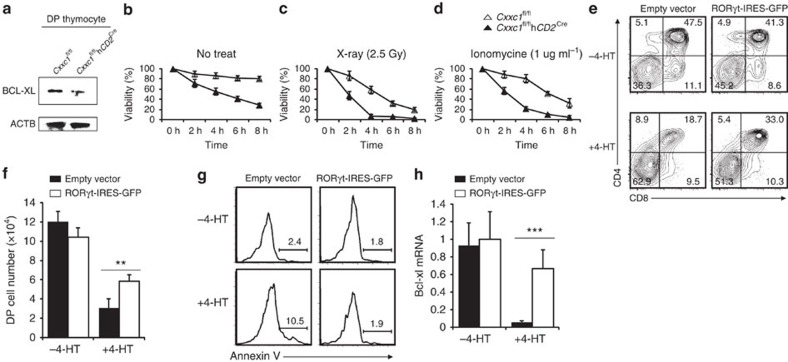
Cxxc1-regulated RORγt partially reversed the survival defects in *Cxxc1*-deficient DP thymocytes. (**a**) DP thymocyte lysates from WT and *Cxxc1*-deficient mice were subjected to SDS–PAGE and blotted with anti-BCL-XL and anti-ACTB. Data comes from three distinct sorted DP cell sets (each from two mice). (**b**–**d**) *In vitro* apoptosis assays. Fluorescence-activated cell sorting-sorted DP thymocytes from indicated mice were cultured with indicated treatment in 48 wells (untreated) (**b**), 2.5 Glay X-ray (**c**) or 1 μg ml^−1^ ionomycine (**d**), and collected at indicated time points and stained by annexin V. Data are from three independently sorted DP cell sets (each from three mice). (**e**–**h**) DN3 cells sorted from *Cxxc1*^fl/fl^CreERT2^+^ mice were seeded onto OP9-DL1 cell layer for 36 h at presence or absence of 4-hydoxytamoxifen (4-HT) and then were transfected with indicated retrovirus. After additional 5 days, cells were analysed by staining with anti-CD4 and anti-CD8a antibodies, and stained by annexin V. Analysis of CD4 versus CD8 of GFP^+^ thymocytes (**e**); the total number of thymocytes in co-culture system (**f**); the annexin V staining of GFP^+^ DP thymocytes (**g**); and mRNA expression Bcl-xl (detected by qPCR with reverse transcription) in GFP^+^ DP thymocytes (**h**) were shown. Results come from three-independent mice experiments. The statistical significance was determined by unpaired *t*-test (two tailed). **P<0.01, ***P<0.001. Error bars indicate s.d.

Rorc, another important Cxxc1 target, encoding the RORγt protein in T cells, functions to maintain DP thymocyte survival through regulating anti-apoptotic gene Bcl-xl^32^,^33^. Our data showed a significant decline in the protein levels of RORγt (Fig. 4h) and BCL-XL (Fig. 6a) in *Cxxc1*-deficient thymocytes. Thus, we speculated that the impaired survival of *Cxxc1*-deficient DP thymocytes was likely to account for the markedly reduced thymic cellularity. Indeed, the survival of *Cxxc1*-deficient DP thymocytes was severely diminished (Fig. 6b), exemplified by their compromised ability to survive from X-ray irradiation (Fig. 6c) and ionomycin stimulation (Fig. 6d). Although Bcl2 and Bcl-xl have reciprocal patterns of expression in developing thymocytes, both of them can inhibit thymocyte death^34^. We found that the reduced cellularity in *Cxxc1*-deficient thymus could be partially rescued by the ectopic expression of BCL2 in the hBcl2 transgenic mice (Supplementary Fig. 8b). In addition, the loss of DP and CD8 SP, as well as the survival of DP thymocytes could be reversed by ectopic expression of BCL2 (Supplementary Fig. 8c–e). The remarkable increase of *Cxxc1*-deficient DP thymocyte apoptosis, indicated by annexin V staining, could also be corrected by the ectopic expressed BCL2 (Supplementary Fig. 8f). These data suggest that the survival defects of *Cxxc1*-deficient DP thymocytes can be partially rescued by anti-apoptotic genes such as Bcl2.

Then, we asked whether RORγt could reverse the survival defects in *Cxxc1*-deficient thymocytes using the *Cxxc1*^fl/fl^CreERT2^+^ and OP9-DL1 cell co-culture system. Ectopic expression of RORγt in *Cxxc1*-sufficient cells (−4-HT) had no significant impact on DP differentiation (Fig. 6e). In contrast, ectopic expression of RORγt remarkably improved the number and frequency of *Cxxc1*-deficient DP cells (+4-HT) (Fig. 6e,f). Moreover, RORγt reversed the *Cxxc1*-deletion-induced apoptosis of DP cells (Fig. 6g) and significantly elevated the expression of Bcl-xl in *Cxxc1*-deficient thymocytes (Fig. 6h). These data strongly support that Cxxc1 controls DP thymocyte survival through regulating RORγt.

## Discussion

Epigenetic regulation is an essential mechanism to coordinate intrathymic T-cell development. Here we reported that Cxxc1, an important component of the H3K4 methyltransferase complex Setd1, directly regulated the promoter-associated H3K4me3 and expression of genes that are essential for thymocyte survival and TCR signalling. Deficiency of *Cxxc1* led to an almost complete block of transition from DN to DP stage in the thymus, suggesting that Cxxc1 is required for thymocyte development.

Our understanding of how epigenetic enzymes program thymocyte development has been significantly advanced in the past decade. Lee *et al*.^5^ has shown that *Lck*^Cre^-mediated deletion of *Dnmt1* in the thymus leads to 85% reduction of total thymocytes and over 90% reduction of DP and CD4^+^ SP thymocytes. On the other hand, deletion of the histone methyltransferase Ezh2 and G9a in T cells has minimal impact on thymocyte development^35^,^36^. In addition, deletion of H3K27me3 demethylases *Jmjd3* and *Utx* by *CD4*^Cre^ only leads to minor changes in thymocyte subpopulations^13^, while deletion of a component of Hbo1 histone acetyltransferase *Brd1* does not change the total number of thymocytes, but significantly decreases CD8^+^ expression in thymocytes^12^. Deletion of *Hdac1*, *Hdac2* or *Hdac7* alone has no impact on thymocyte development, whereas only double deletion of both *Hdac1* and *Hdac2* leads to fivefold reduction in thymocyte cellularity^8^,^10^. In addition, *Lck*^Cre^-mediated deletion of mixed-lineage leukemia 1 (Mll1), the key member of MLL histone methyltransferase family, does not interrupt normal T-cell development in the thymus^37^. However, our data showed that the loss of *Cxxc1* led to 99% reduction in the thymic cellularity, with very few DP left and almost 100% depletion of SP cells (Fig. 1a). The peripheral CD4^+^ and CD8^+^ T cells were also abolished due to inability of thymocyte development (Fig. 1d). These data suggest that Cxxc1 plays a non-redundant indispensable role during T-cell development in the thymus.

Cxxc1 functions through regulating of DNA methylation and/or H3K4me3 in ES cells^17^. It is vital to clarify whether it also plays dual roles in thymocyte development. Our data demonstrated that Cxxc1 controlled T-cell differentiation by modifying the promoter-associated H3K4me3 levels (Figs 2a,b; 3e), which in turn led to broad gene expression regulation in DP thymocytes (Fig. 3g,h; Supplementary Fig. 5c). However, its role in regulating DNA methylation was not essential for the appropriate thymocyte development (Fig. 2a,d; Supplementary Fig. 4e). Consistently, a recent report also suggests *Mx1*^Cre^-mediated Cxxc1 deletion has no effect on DNA methylation in haematopoietic stem cells^21^. Therefore, unlike Cxxc1's role in ES cells, it may only function as an H3K4me3 regulator in haematopoietic cells such as T cells.

Previous study has identified a significant overlap among genome-wide Cxxc1-binding sites, H3K4me3 marks and CpGI in brain tissues^18^. Considering the mixed nature of multiple cell types in brain tissues, the Cxxc1 binding and H3K4me3 profiles can be treated as a collection of all possible DNA loci associated with Cxxc1 or H3K4me3 modification disregarding the cell type and tissue specificity. However, it is unclear whether Cxxc1 colocalizes with H3K4me3 and/or CpGI in a single type of cells. Our data indicated that ∼6% of Cxxc1-binding sites were colocalized with H3K4me3 and CpGI in DP thymocytes (Fig. 3d,f). In addition, only 12% of the Cxxc1-binding sites in DP thymocytes were also identified in brain tissues (Supplementary Fig. 5b). These data suggest a profound difference in Cxxc1-mediated epigenetic program between neuronal tissue and immune cells. Similarly, previous studies have also reported the difference of Cxxc1 functions in multiple types of cells^17^,^22^,^38^,^39^. Therefore, our data illustrates that a cell-type-specific binding profile of Cxxc1 may decide its primary function and targeting Cxxc1 for future epigenetic therapy that may achieve cell-type-specific drug effects with limited complications.

RORγt promotes survival of thymocyte through maintaining the expression of Bcl-xl^40^,^41^. In our study, we found Cxxc1 directly bound to Rorc gene locus (Fig. 4a,b) and coordinated histone modifications (including H3K4me3, H3K9ac, and H3K27ac; Fig. 4d–f) to maintain active expression of RORγt in thymocytes (Fig. 4g,h). Deficiency of *Cxxc1* led to the loss of Rorc gene expression as well as profound DP thymocyte survival defects, accompanying the reduction in BCL-XL expression (Fig. 6a). Importantly, our data also showed that RORγt partially rescued the survival defects caused by *Cxxc1* deficiency, suggesting that Cxxc1 is required for thymocyte survival through regulating RORγt expression (Fig. 6e–g). In DP thymocytes, Wnt–βcatenin–TCF-1 pathway and HeLa E-box-binding factor (HEB) are responsible for maintaining RORγt expression and T-cell survival^42^,^43^,^44^. However, it is yet to be defined how the Cxxc1 and Wnt signalling pathway work in concert to regulate RORγt expression in DP thymocytes.

## Methods

### Mice

Mice carrying the floxed *Cxxc1* allele were generated by homologous recombination-mediated gene targeting in ES cells of strain 129 at the Shanghai Research Center for Model Organisms. Cxxc1-targeted (*Cxxc1*^*T*^) mice were generated by homologous recombination-mediated gene targeting in ES cells of strain 129 (ref. 45), strategy was shown in Supplementary Fig. 2a. The *Cxxc1*^*T*^mice were crossed with FLPeR mice to delete the Neo cassette and gain the *Cxxc1*^fl/fl^ mice. The *Cxxc1*^fl/fl^ mice were then backcrossed onto the C57BL/6 background for five generations. Exons 4 and 5 in *Cxxc1* were deleted by Cre recombinase and a new stop code was generated at the downstream of exon 3 in *Cxxc1* gene. To obtain the *Cxxc1*^+/−^ mice, the *Cxxc1*^T^ mice were crossed with the PGK^Cre^ mice, which resulted in widespread deletion of *Cxxc1* (ref. 46). The *Cxxc1*^+/−^ is normal and fertile; however, no *Cxxc1*^−/−^ mice were born from *Cxxc1*^+/−^ crossing (Supplementary Fig. 2b). The primers for Cxxc1 detecting the homologous recombination arm, loxp site and deleted band in Cxxc1 locus are listed in Supplementary Table 3.

The FLPeR (JAX: 003946), *PGK*^Cre^ (JAX: 020811), Lck^Cre^ (JAX: 006889), *hCD2*^Cre^ (JAX: 008520) mice, the OTI (JAX: 003831) and OTII (JAX: 004194) TCR transgenic mice were from Jackson Laboratory. The CreERT2^+^ mice^47^ were gifts from Dr Y.W. He (Duke University Medical Center). The Bcl2 transgenic mice^48^ were gifts from Dr J. Adams (The Walter and Eliza Hall Institute of Medical Research). All experiments were performed with 3–4-week-old mice unless specified. All mice were kept in the Zhejiang University Laboratory Animal Center and all animal experimental procedures were approved by the Animal Review Committee at Zhejiang University School of Medicine.

### Cell culture and retrovirus transfection

OP9 bone marrow stromal cells expressing the Notch ligand DL-1 (OP9-DL1), provided kindly by Dr J.C. Zúñiga-Pflücker (University of Toronto, Toronto, Canada), were cultured and maintained in α-MEM (Gibco) medium with 10% fetal bovine serum (Gibco). For DN3 cells co-cultures, the sorted DN3 cells were plated onto confluent OP9-DL1 monolayers (70–80% confluent) and cultured with of 5 ng ml^−1^ recombinant murine interleukin-7 (Peprotech) and 5 ng ml^−1^ Flt3L (Peprotech).

Retrovirus preparation was performed in Plat-E cells. Plat-E cells were transfected with pMX-IRES-GFP containing indicated genes, the medium were replaced with fresh medium after 10 h, and retrovirus supernatant was collected after additional 72 h.

Expression of ectopic proteins in DN3 cells was performed as previously described^49^. In brief, RetroNectin (Takara) coating and washing were carried out according to the manufacturer's instruction. The retrovirus was added to wells coated with RetroNectin, followed by 4 h incubation at room temperature and removal of the retrovirus. After 24 h co-culture, DN3 cells were directly placed on plates coated with RetroNectin and retrovirus. And trypsinized OP9-DL1 cells were added to the DN3 cells.

### Assay of DP development

Purified DP thymocytes were cultured in RPMI-1640 medium overnight with plate-bound anti-TCRβ (H57; BioLegend, 10 μg ml^−1^) and anti-CD2 (RM2-5; BD Bioscience, 10 μg ml^−1^) antibodies as previously described^50^. Then cells were washed and analysed immediately by flow cytometry (stimulatory culture) or cultured further for 24 h in the same medium (recovery culture)

### Apoptosis assay

Purified DP thymocytes were resuspended in DMEM medium with 10% fetal bovine serum, and cultured under indicated conditions. At the indicated time points, cells were collected and analysed by flow cytometry for viability using annexin fluorescein isothiocyanate (V-FITC) apoptosis detection kit (BioLegend, 640914).

### Flow cytometry and antibodies

The following eBiosciences antibodies were used in our experiments: CD4 (RM4-5, 1 μg ml^−1^), CD8a (53-6.7, 2.5 μg ml^−1^), CD8b (H35-17.2, 2.5 μg ml^−1^), CD25 (PC61, 1 μg ml^−1^), CD44 (IM7, 1 μg ml^−1^), TCRβ (H57-597, 1 μg ml^−1^), NK1.1 (PK136, 1 μg ml^−1^), CD24 (M1/69, 1 μg ml^−1^), TCRγδ (GL3, 1 μg ml^−1^), CD5(53-7.3, 1 μg ml^−1^), CD69 (H1.2F3, 1 μg ml^−1^), CD62L (MEL-14, 1 μg ml^−1^), Vα2 (B20.1, 1 μg ml^−1^), Vβ5 (MR9-4, 1 μg ml^−1^), CD11b (M1/70, 1 μg ml^−1^), CD11c (N418, 1 μg ml^−1^), CD122 (TM-b1, 1 μg ml^−1^), Gr-1 (RB6-8C5, 1 μg ml^−1^), Ter119 (TER-119, 1 μg ml^−1^), CD19 (eBio1D3, 1 μg ml^−1^), Zap70 (1E7.2, 2.5 μg ml^−1^) and RORγt (B2D, 2.5 μg ml^−1^). Allophycocyanin (APC) annexin V (1:20 dilution) and Brilliant Violet 421 anti-rabbit IgG (Poly4064, 1:50 dilution) were from BioLegend. Flow cytometry was performed with FACSCalibur and FACSAria II machines (BD Biosciences). Data were analysed by FlowJo software (Tree Star, Inc.). Intracellular staining was processed using IC fixation buffer (eBiosciences), Foxp3/transcription factor staining buffer set (eBiosciences) was used for RORγt and DNMT1 staining. Cells were sorted by a FACSAria II flow cytometer. DN cells were sorted and analysed by staining thymocyte with the following antibodies, PE-NK1.1, PE-CD11c, PE-CD11b, PE-TER119, PE-TCRγδ, PE-CD122, PE-CD19 and PE-Gr-1 to exclude non-DN cells. And dead cells were excluded by 4,6-diamidino-2-phenylindole staining. DN3 cells (0.3 million) were used for each co-culture with OP9-DL1.

Anti-Bcl-xl (2H12, 1:1,000 dilution), anti-LCK (ab3885, 1:1,000 dilution), anti-DNMT1 (ab13537, 1:100 dilution) and anti-Setd1 (ab70378, 1:1,000 dilution for western blot; 4 μg for each IP reaction) were from Abcam. Anti-JNK (2C6, 1:1,000 dilution), anti-p-JNK (Thr183/Tyr185; G9, 1:1,000 dilution) were from Cell Signaling Technology. Anti-CXXC1 (H-120, 1:200 dilution for western blot; 4 μg for each IP and ChIP reaction), anti-ERK (C-9, 1:1,000 dilution) and anti-p-ERK (E-4, 1:1,000 dilution) were from Santa Cruz Biotechnology.

### ChIP and data analysis

ChIP assays were performed according to the manufacturers' instructions with modifications using the ChIP-IT kit (Active Motif, USA). In brief, the DP thymocytes were fixed by 1% formaldehyde. The crosslinked chromatin was sonicated in a 4 °C water bath using Bioruptor UCD-200 sonicator to obtain DNA fragments sized between 100 and 500 bp. Chromatin from 2 × 10^6^ cells was used for each ChIP experiment. Antibodies against Cxxc1, H3K4me3 (Active Motif, 4 μg for each ChIP reaction), H3K27me3, H3K9me2, H3K9ac and H3K27ac (Abcam, 4 μg for each ChIP reaction) were used. The ChIP qPCR primers were listed in Supplementary Table 4. The immunoprecipitated DNA was purified and subjected to sequencing library preparation using the KAPA HTP Library Preparation Kit (Kapa Biosystems, USA) according to the manufacture's protocol. The DNA libraries were then sequenced with Illumina HiSeq 2500 at the Zhongshan Ophthalmic Center core facility.

Sequenced reads of 50 bp were obtained using the CASAVA 1.8.2 package (Illumina). All reads were mapped to the mouse genome mm9 and those uniquely mapped reads were subjected to further peak identification process. SICER_V1.1 was used to identify significant peaks (FDR=10^−5^) with both input DNA and ChIP DNA in *Cxxc1*-deficient cells as controls. Output of the peak files was converted to browser-extensible data files and viewed with UCSC genome browser. The MEME-ChIP (Machanick and Bailey, 2011) was used to perform the consensus binding motif analysis for Cxxc1. To calculate the tag density of Cxxc1 binding or H3K4me3 modifications around TSS or the centres of CpGI, uniquely mapped tags were summarized in 100-bp windows, and all window tag counts were normalized by the total number of bases in the windows and the total read number of the given sample.

### RNA-seq and data analysis

Total RNA was extracted from WT and *Cxxc1*-deficient DP thymocytes with TRIzol reagent (Invitrogen, USA). mRNA-seq libraries were prepared using NEBNext mRNA Library Prep Master Mix Set for Illumina according to the manufacture's protocol (NEB, USA). All libraries were then sequenced with Illumina HiSeq 2500 at the Zhongshan Ophthalmic Center core facility. Sequenced reads of 50 bp were obtained using the CASAVA 1.8.2 package (Illumina). All reads were mapped to the mouse genome mm9 and those uniquely mapped reads were subjected to RNA-seq data analysis using Partek Genomic Suite 6.6 (Partek, USA).

### Histone H3 methylated Lys ELISA

The ELISA was performed with the H3K4me3 detection kit from Active Motif (53101). The crude histone protein was prepared using acid extract method. The extracted crude histone protein was subjected to ELISA according to the manufacturer's instruction.

### Total 5mC detection by dot blotting of genomic DNA

Genomic DNA was extracted from DP thymocytes (adjusted to the same concentration), then denatured at 99 °c for 10 min, followed by immediately incubation on ice. The single-strand DNA was bound to a nitrocellulose membrane, air dried and crosslinked with ultraviolet light. The membrane was subjected to immunoblotting using anti-5mC antibodies (Calbiochem, NA81, 1:1,000 dilution) followed by horseradish peroxidase-conjugated secondary antibodies staining.

### RNA isolation and real-time qPCR

TRIzol reagent (Invitrogen) was used for total RNA extraction. Real-time qPCR was performed using SYBR Premix Ex TaqTM II on LC480II real-time PCR system (Roche, USA). Results were normalized to GAPDH expression. Primers for qPCR are listed in Supplementary Table 5.

### Plasmid construction

Recombinant vector encoding murine RORγt and the constructs with full-length and truncated/muted Cxxc1 genes was constructed by PCR-based amplification and subcloning into the pMX-IRES-GFP vector.

### Methylated DNA immunoprecipitation

MeDIP assays were performed according to the manufacturers' instruction (Active Motif 55009). In brief, DNA extracted from indicated populations of WT mice (Zymo Research D3024) was sonicated in a 4 °C water bath using Bioruptor UCD-200 sonicator to obtain DNA fragments sized between 100 and 500 bp. Then sonicated DNA was denatured at 95 °C for 10 min, followed by incubation on ice. And the IP reaction was performed using anti-5mC antibody. The immunoprecipitated DNA was purified and subjected to qPCR analysis. The primers were listed in Supplementary Table 6.

### Immunoprecipitation and western blot

A measure of 50 μl protein G breads (Invitrogen) were pre-incubated with antibodies for 4 h. Cells were lysed in Triton X-100 lysis buffer containing 50 mM Tris (pH 7.4), 300 mM NaCl, 0.5% Triton X-100, phenylmethylsulfonyl fluoride (PMSF) and protease inhibitor 'cocktail' (Roche). Cell lysates were immunoprecipitated overnight at 4 °C with the antibody-conjugated beads. After being washed four times, samples were resolved by SDS–polyacrylamide gel electrophoresis (PAGE) gels (10%) and blotted. For immunoblot analysis, sorted cells were lysed in complete lysis-M buffer (Roche; cat. no. 04719956001), and total protein was subjected to SDS–PAGE, transferred onto nitrocellulose membrane and hybrid-blotted. Densitometry was determined by Quantity One software. Images have been cropped for presentation. The full-size images are presented in the Supplementary Fig. 9.

### Deletion of Cxxc1 in *Cxxc1* ^fl/fl^CreERT2 cells

Sorted DN3 cells from Cxxc1fl/flCreERT2 mice thymocytes were co-cultured with OP9-DL1 monolayers at presence of 200 nm 4-hydroxtamoxifen treatment.

### Statistical analysis

Statistical analyses were performed using Excel except for RNA-seq and ChIPseq data. Bars in graphs indicate s.d, except where otherwise indicated, comparisons were performed by two-tailed unpaired Student's *t*-test. Significance levels (*P* values) are presented on figures.

### Data availability

The ChIP-seq and RNA-seq data sets were deposited in gene expression omnibus (GEO), with an accession number of GSE79976, are available via the repository's data access request procedures.

## Additional information

**How to cite this article**: Cao, W. *et al*. CXXC finger protein 1 is critical for T-cell intrathymic development through regulating H3K4 trimethylation. *Nat. Commun*. 7:11687 doi: 10.1038/ncomms11687 (2016).

## Supplementary Material

Supplementary Information Supplementary Figures 1-9, Supplementary Tables 1 -3

Supplementary Dataset 1 gene expression data

## References

[b1] Rothenberg E. V. Transcriptional drivers of the T-cell lineage program. Curr. Opin. Immunol. 24, 132–138 (2012). 22264928 10.1016/j.coi.2011.12.012 PMC3319509

[b2] Rothenberg E. V., Moore J. E. & Yui M. A. Launching the T-cell-lineage developmental programme. Nat. Rev. Immunol. 8, 9–21 (2008). 18097446 10.1038/nri2232 PMC3131407

[b3] Singer A. & Bosselut R. CD4/CD8 coreceptors in thymocyte development, selection, and lineage commitment: analysis of the CD4/CD8 lineage decision. Adv. Immunol. 83, 91–131 (2004). 15135629 10.1016/S0065-2776(04)83003-7

[b4] Singer A., Adoro S. & Park J.-H. Lineage fate and intense debate: myths, models and mechanisms of CD4- versus CD8-lineage choice. Nat. Rev. Immunol. 8, 788–801 (2008). 18802443 10.1038/nri2416 PMC2760737

[b5] Lee P. P. . A critical role for Dnmt1 and DNA methylation in T cell development, function, and survival. Immunity 15, 763–774 (2001). 11728338 10.1016/s1074-7613(01)00227-8

[b6] Sellars M. . Regulation of DNA methylation dictates Cd4 expression during the development of helper and cytotoxic T cell lineages. Nat. Immunol. 16, 746–754 (2015). 26030024 10.1038/ni.3198 PMC4474743

[b7] Zhang J. A., Mortazavi A., Williams B. A., Wold B. J. & Rothenberg E. V. Dynamic transformations of genome-wide epigenetic marking and transcriptional control establish T cell identity. Cell 149, 467–482 (2012). 22500808 10.1016/j.cell.2012.01.056 PMC3336965

[b8] Dovey O. M. . Histone deacetylase 1 and 2 are essential for normal T-cell development and genomic stability in mice. Blood 121, 1335–1344 (2013). 23287868 10.1182/blood-2012-07-441949 PMC3836254

[b9] Dequiedt F. . HDAC7, a thymus-specific class II histone deacetylase, regulates Nur77 transcription and TCR-mediated apoptosis. Immunity 18, 687–698 (2003). 12753745 10.1016/s1074-7613(03)00109-2

[b10] Kasler H. G. . Histone deacetylase 7 regulates cell survival and TCR signaling in CD4/CD8 double-positive thymocytes. J. Immunol. 186, 4782–4793 (2011). 21398603 10.4049/jimmunol.1001179

[b11] Stengel K. R. . Histone deacetylase 3 is required for efficient T cell development. Mol. Cell. Biol. 35, 3854–3865 (2015). 26324326 10.1128/MCB.00706-15 PMC4609739

[b12] Mishima Y. . Histone acetylation mediated by Brd1 is crucial for Cd8 gene activation during early thymocyte development. Nat. Commun. 5, 5872 (2014). 25519988 10.1038/ncomms6872 PMC4490789

[b13] Manna S. . Histone H3 Lysine 27 demethylases Jmjd3 and Utx are required for T-cell differentiation. Nat. Commun. 6, 8152 (2015). 26328764 10.1038/ncomms9152 PMC4569738

[b14] Carlone D. L. . Reduced genomic cytosine methylation and defective cellular differentiation in embryonic stem cells lacking CpG binding protein. Mol. Cell. Biol. 25, 4881–4891 (2005). 15923607 10.1128/MCB.25.12.4881-4891.2005 PMC1140577

[b15] Lee J.-H. & Skalnik D. G. CpG-binding protein (CXXC Finger Protein 1) Is a component of the mammalian Set1 histone H3-Lys 4 Methyltransferase complex, the analogue of the yeast Set1 / COMPASS. J. Biol. Chem. 280, 41725–41731 (2005). 16253997 10.1074/jbc.M508312200

[b16] Butler J. S., Lee J.-H. & Skalnik D. G. CFP1 interacts with DNMT1 independently of association with the Setd1 histone H3K4 methyltransferase complexes. DNA Cell. Biol. 27, 533–543 (2008). 18680430 10.1089/dna.2007.0714 PMC2754740

[b17] Tate C. M., Lee J. H. & Skalnik D. G. CXXC finger protein 1 contains redundant functional domains that support embryonic stem cell cytosine methylation, histone methylation, and differentiation. Mol. Cell. Biol. 29, 3817–3831 (2009). 19433449 10.1128/MCB.00243-09 PMC2704740

[b18] Thomson J. P. . CpG islands influence chromatin structure via the CpG-binding protein Cfp1. Nature 464, 1082–1086 (2010). 20393567 10.1038/nature08924 PMC3730110

[b19] Skalnik D. G. CpG Binding protein is crucial for early embryonic development. Mol. Cell. Biol. 21, 7601–7606 (2001). 11604496 10.1128/MCB.21.22.7601-7606.2001 PMC99931

[b20] Young S. R. L., Mumaw C., Marrs J. A. & Skalnik D. G. Antisense targeting of CXXC finger protein 1 inhibits genomic cytosine methylation and primitive hematopoiesis in zebrafish. J. Biol. Chem. 281, 37034–37044 (2006). 17023431 10.1074/jbc.M604546200

[b21] Chun K. T. . The epigenetic regulator CXXC finger protein 1 is essential for murine hematopoiesis. PLoS ONE 9, e113745 (2014). 25470594 10.1371/journal.pone.0113745 PMC4254612

[b22] Clouaire T. . Cfp1 integrates both CpG content and gene activity for accurate H3K4me3 deposition in embryonic stem cells. Genes Dev. 26, 1714–1728 (2012). 22855832 10.1101/gad.194209.112 PMC3418589

[b23] Skalnik D. G. CpG binding protein is crucial for early embryonic development. Mol. Cell. Biol. 21, 7601–7606 (2001). 11604496 10.1128/MCB.21.22.7601-7606.2001 PMC99931

[b24] Zhumabekov T., Corbella P., Tolaini M. & Kioussis D. Improved version of a human CD2 minigene based vector for T cell-specific expression in transgenic mice. J. Immunol. Methods. 185, 133–140 (1995). 7665895 10.1016/0022-1759(95)00124-s

[b25] Mao X., Fujiwara Y. & Orkin S. H. Improved reporter strain for monitoring Cre recombinase-mediated DNA excisions in mice. Proc. Natl Acad. Sci. 96, 5037–5042 (1999). 10220414 10.1073/pnas.96.9.5037 PMC21812

[b26] Li P. . Reprogramming of T cells to natural killer–like cells upon Bcl11b deletion. Science 329, 85–89 (2010). 20538915 10.1126/science.1188063 PMC3628452

[b27] Butler J. S., Lee J.-H. & Skalnik D. G. CFP1 interacts with DNMT1 independently of association with the Setd1 histone H3K4 methyltransferase complexes. DNA Cell. Biol. 27, 533–543 (2007). 18680430 10.1089/dna.2007.0714 PMC2754740

[b28] Huang, da W., Sherman B. T. & Lempicki R. A. Systematic and integrative analysis of large gene lists using DAVID bioinformatics resources. Nat. Protoc. 4, 44–57 (2009). 19131956 10.1038/nprot.2008.211

[b29] Suzuki H., Punt J. A., Granger L. G. & Singer A. Asymmetric signaling requirements for thymocyte commitment to the CD4^+^ versus CD8^+^ T cell lineages: a new perspective on thymic commitment and selection. Immunity 2, 413–425 (1995). 7719943 10.1016/1074-7613(95)90149-3

[b30] Lucas B. & Germain R. N. Unexpectedly complex regulation of CD4/CD8 coreceptor expression supports a revised model for CD4^+^CD8^+^ thymocyte differentiation. Immunity 5, 461–477 (1996). 8934573 10.1016/s1074-7613(00)80502-6

[b31] Brugnera E. . Coreceptor Reversal in the thymus: signaled CD4^+^8^+^ thymocytes initially terminate CD8 transcription even when differentiating into CD8^+^ T cells. Immunity 13, 59–71 (2000). 10933395 10.1016/s1074-7613(00)00008-x

[b32] Sun Z. . Requirement for RORγ in thymocyte survival and lymphoid organ development. Science 288, 2369–2373 (2000). 10875923 10.1126/science.288.5475.2369

[b33] Xi H., Schwartz R., Engel I., Murre C. & Kersh G. J. Interplay between RORγt, Egr3, and E proteins controls proliferation in response to pre-TCR signals. Immunity 24, 813–826 (2006). 16782036 10.1016/j.immuni.2006.03.023

[b34] Chao D. T. . Bcl-XL and Bcl-2 repress a common pathway of cell death. J. Exp. Med. 182, 821–828 (1995). 7650488 10.1084/jem.182.3.821 PMC2192158

[b35] Tumes D. J. . The polycomb protein Ezh2 regulates differentiation and plasticity of CD4^(+)^ T helper type 1 and type 2 cells. Immunity 39, 819–832 (2013). 24238339 10.1016/j.immuni.2013.09.012

[b36] Lehnertz B. . Activating and inhibitory functions for the histone lysine methyltransferase G9a in T helper cell differentiation and function. J. Exp. Med. 207, 915–922 (2010). 20421388 10.1084/jem.20100363 PMC2867284

[b37] Jude C. D. . Unique and independent roles for MLL in adult hematopoietic stem cells and progenitors. Cell Stem Cell 1, 324–337 (2007). 18371366 10.1016/j.stem.2007.05.019 PMC2234224

[b38] Clouaire T., Webb S. & Bird A. Cfp1 is required for gene expression-dependent H3K4 trimethylation and H3K9 acetylation in embryonic stem cells. Genome Biol. 15, 451 (2014). 25201068 10.1186/s13059-014-0451-x PMC4189735

[b39] Tang Z. . SET1 and p300 act synergistically, through coupled histone modifications, in transcriptional activation by p53. Cell 154, 297–310 (2013). 23870121 10.1016/j.cell.2013.06.027 PMC4023349

[b40] Littman D. R. . Role of the nuclear hormone receptor ROR gamma in transcriptional regulation, thymocyte survival, and lymphoid organogenesis. Cold Spring Harb. Symp. Quant. Biol. 64, 373–381 (1999). 11232310 10.1101/sqb.1999.64.373

[b41] Xi H., Schwartz R., Engel I., Murre C. & Kersh G. J. Interplay between RORgammat, Egr3, and E proteins controls proliferation in response to pre-TCR signals. Immunity 24, 813–826 (2006). 16782036 10.1016/j.immuni.2006.03.023

[b42] Ioannidis V., Beermann F., Clevers H. & Held W. The [beta]-catenin-TCF-1 pathway ensures CD4^+^CD8^+^ thymocyte survival. Nat. Immunol. 2, 691–697 (2001). 11477404 10.1038/90623

[b43] D'Cruz L. M., Knell J., Fujimoto J. K. & Goldrath A. W. An essential role for the transcription factor HEB in thymocyte survival, Tcra rearrangement and the development of natural killer T cells. Nat. Immunol. 11, 240–249 (2010). 20154672 10.1038/ni.1845 PMC2993240

[b44] Yuan J., Crittenden R. B. & Bender T. P. c-Myb promotes the survival of CD4^+^CD8^+^ double-positive thymocytes through upregulation of Bcl-xL. J. Immunol. 184, 2793–2804 (2010). 20142358 10.4049/jimmunol.0902846 PMC2856624

[b45] Liu P., Jenkins N. A. & Copeland N. G. A highly efficient recombineering-based method for generating conditional knockout mutations. Genome Res. 13, 476–484 (2003). 12618378 10.1101/gr.749203 PMC430283

[b46] Lallemand Y., Luria V., Haffner-Krausz R. & Lonai P. Maternally expressed PGK-Cre transgene as a tool for early and uniform activation of the Cre site-specific recombinase. Transgenic Res. 7, 105–112 (1998). 9608738 10.1023/a:1008868325009

[b47] Jia W. & He Y.-W. Temporal regulation of intracellular organelle homeostasis in T lymphocytes by autophagy. J. Immunol. 186, 5313–5322 (2011). 21421856 10.4049/jimmunol.1002404

[b48] Ogilvy S. . Constitutive Bcl-2 expression throughout the hematopoietic compartment affects multiple lineages and enhances progenitor cell survival. Proc. Natl Acad. Sci. USA 96, 14943–14948 (1999). 10611317 10.1073/pnas.96.26.14943 PMC24752

[b49] Kreslavsky T. . β-selection-induced proliferation is required for αβ T cell differentiation. Immunity 37, 840–853 (2012). 23159226 10.1016/j.immuni.2012.08.020 PMC3709258

[b50] Cibotti R., Punt J. A., Dash K. S., Sharrow S. O. & Singer A. Surface molecules that drive t cell development in vitro in the absence of thymic epithelium and in the absence of lineage-specific signals. Immunity 6, 245–255 (1997). 9075925 10.1016/s1074-7613(00)80327-1

