# Supplementary material for: CXXC finger protein 1 is critical for T-cell intrathymic development through regulating H3K4 trimethylation
Source: Nat Commun. 2016 May 23;7:11687. doi: 10.1038/ncomms11687 (PMC4879243; doi:10.1038/ncomms11687)
Supplement: Supplementary Information — Supplementary Figures 1-9, Supplementary Tables 1 -3 [file ncomms11687-s1.pdf]

Supplemental Figures

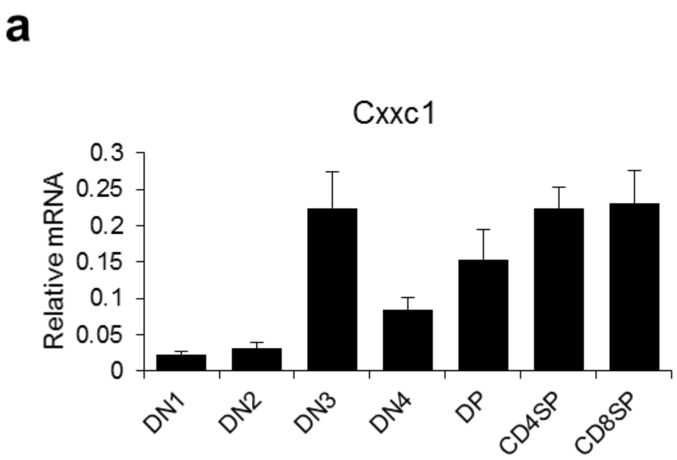

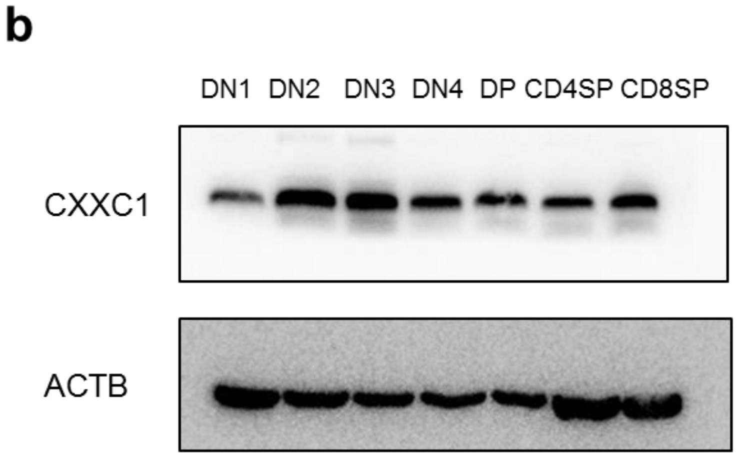

Supplementary Figure 1

Profile of Cxxc1 expression during T cell development

(a) Quantification of Cxxc1 mRNA expression in different stages. Indicated subpopulations were sorted from wild-type mice and used for Real-time Quantitative RT-PCR analysis. Error bars indicate s.d.
(b) Detection of Cxxc1 protein in different stages, Indicated subpopulations were sorted from wild-type mice and used for Western Blot analysis. Data comes from three independently sorted samples (for mRNA detection, 3 mice used; for protein detection, 6 mice used).

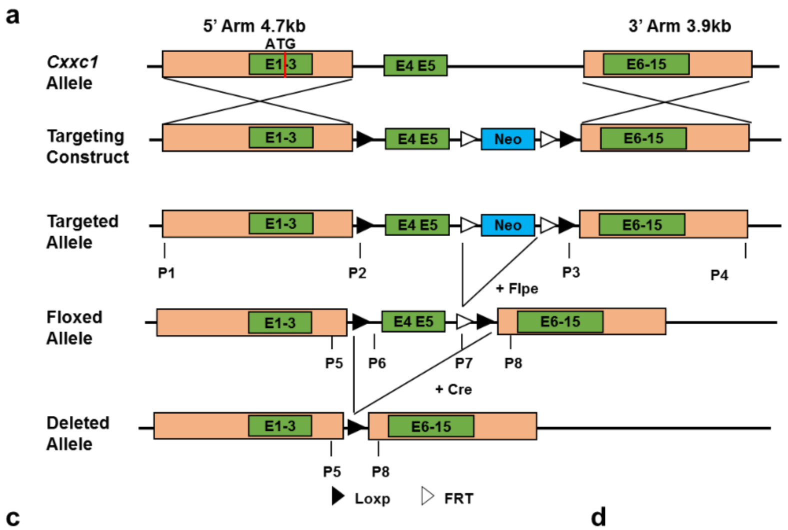

b

Cxxc1$^{+/-}$ X *Cxxc1*$^{+/-}$

Parent Pair	Number			Total
	+/+	+/-	-/-	
1	2	4	0	6
2	6	7	0	13
3	3	8	0	11

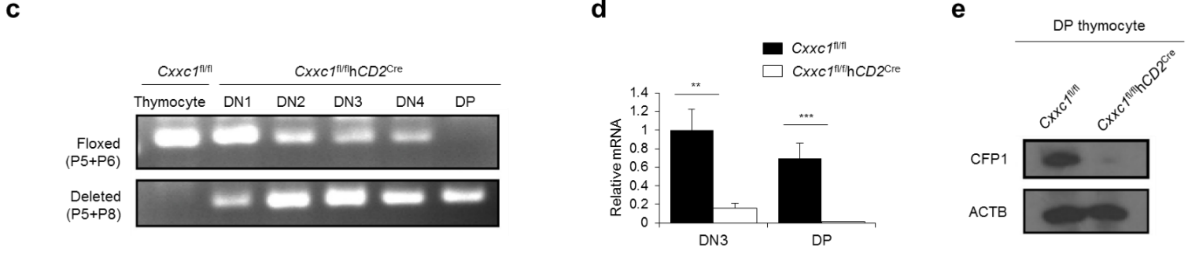

Supplementary Figure 2

Conditional targeting of the mouse *Cxxc1* gene

(a) Strategy of Cxxc1 conditional knockout. Primers P1 and P2 for 5' arm homologous recombination detection, P3 and P4 for 3' arm homologous recombination detection, P5 plus P6 and P7 plus P8 for 5' loxped and 3' loxped site detection respectively, P5 plus P8 for deleted allele detection. (b) Genotyping of offspring from *Cxxc1*$^{+/-}$ X *Cxxc1*$^{+/-}$. (c) Genomic DNA were isolated from subsets of thymocytes from indicated mice and then used for PCR analysis of *Cxxc1* deletion and the Loxp-flanked allele. Data comes from three distinct sorted samples sets (each from 2 mice).(d) Quantification of Cxxc1 mRNA expression in DN3 and DP Thymocytes. The indicated cells from *Cxxc1*-deficient and control mice were used for Real-time Quantitative RT-PCR analysis. Data is presented as mean ± SD and from three mice experiments. The statistical significance was calculated by unpaired t-test (two-tailed). $^{**}P < 0.01$; $^{***}P < 0.001$. (e) Detection of CXXC1 protein deletion. DP thymocytes from *Cxxc1*-deficient and control mice were used for western blot analysis. Data is from three DP cell sets (each from 2 mice).

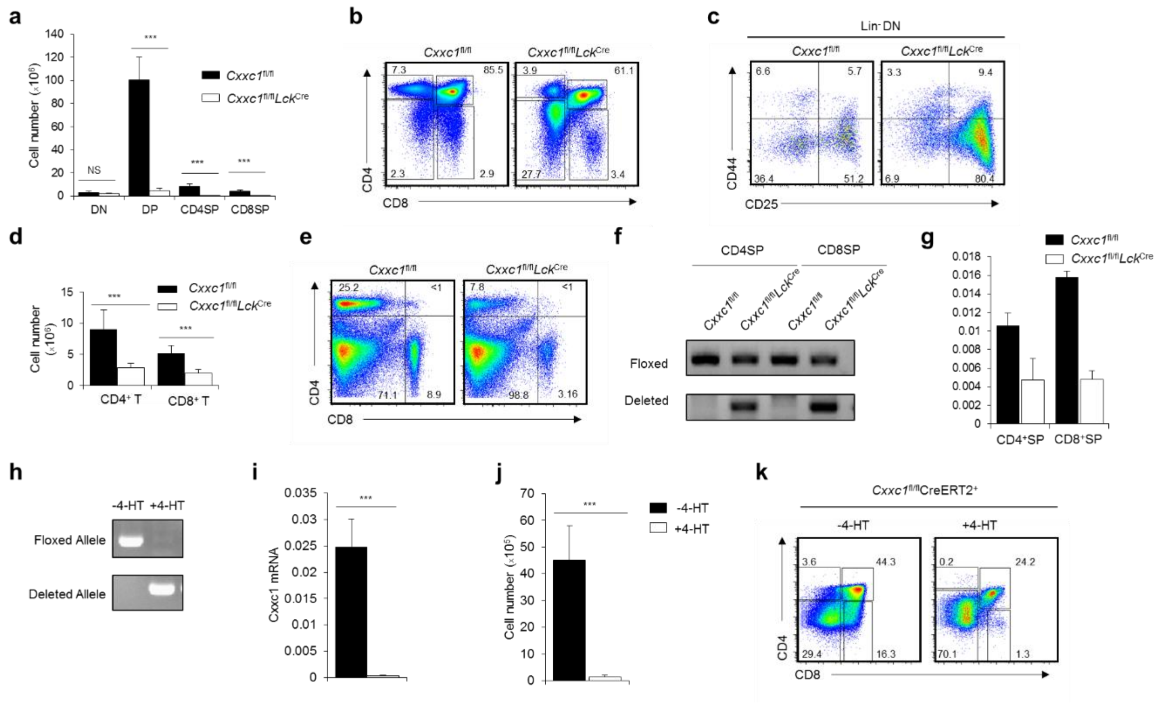

Supplementary Figure 3

Intrathymic T cell development was severely impaired in *Cxxc1* deficient mice

(a) Quantification of DN, DP, CD4+ or CD8+ SP thymocytes subpopulations. The P value was calculated by unpaired t-test (two-tailed). NS, not significant. ***$P < 0.001$. Error bars present s.d. (b) Thymocytes from *Cxxc1*$^{fl/fl}$ or *Cxxc1*$^{fl/fl}$ *Lck*^{Cre} mice were analyzed. (c) Lineage negative cells were analyzed for the profile of DN1-DN4 subsets. (d) Quantitation of the absolute cell numbers of $CD4^+$ and $CD8^+$ T cell subsets, as the product of total splenocytes multiplied by the percentage of cells found in that population. The statistical significance was calculated by unpaired t-test (two-tailed). NS, not significant. ***$P < 0.001$. (e)Total splenocytes from indicated mice were analyzed for CD4 and CD8 expression by flow cytometry. Data is from six mice analysis (a-e). (f, g) $CD4^+$ SP or $CD8^+$ SP cells sorted from indicated mice were used for genome DNA PCR analysis using indicated primers (f); or were used for Real-time Quantitative PCR analysis (g). Data comes from independently sorted samples sets (each from 2 mice). Error bars indicate s.d.(h-k) DN3 cells sorted from *Cxxc1*$^{fl/fl}$ CreERT2$^+$ mice were co-cultured with OP9-DL1 at presence or absence of 4-hydroxytamoxifen (4-HT) for 5 days. PCR analysis of *Cxxc1* deletion and the Loxp-flanked allele (f); detection of Cxxc1 expression by Real-time Quantitative PCR assays (g); the number of thymocytes (h); and flow cytometry analysis of thymocytes (i) were shown. Data is from four mice experiments. The statistical significance was calculated by unpaired t-test (two-tailed). ***$P < 0.001$. Error bars indicate s.d.

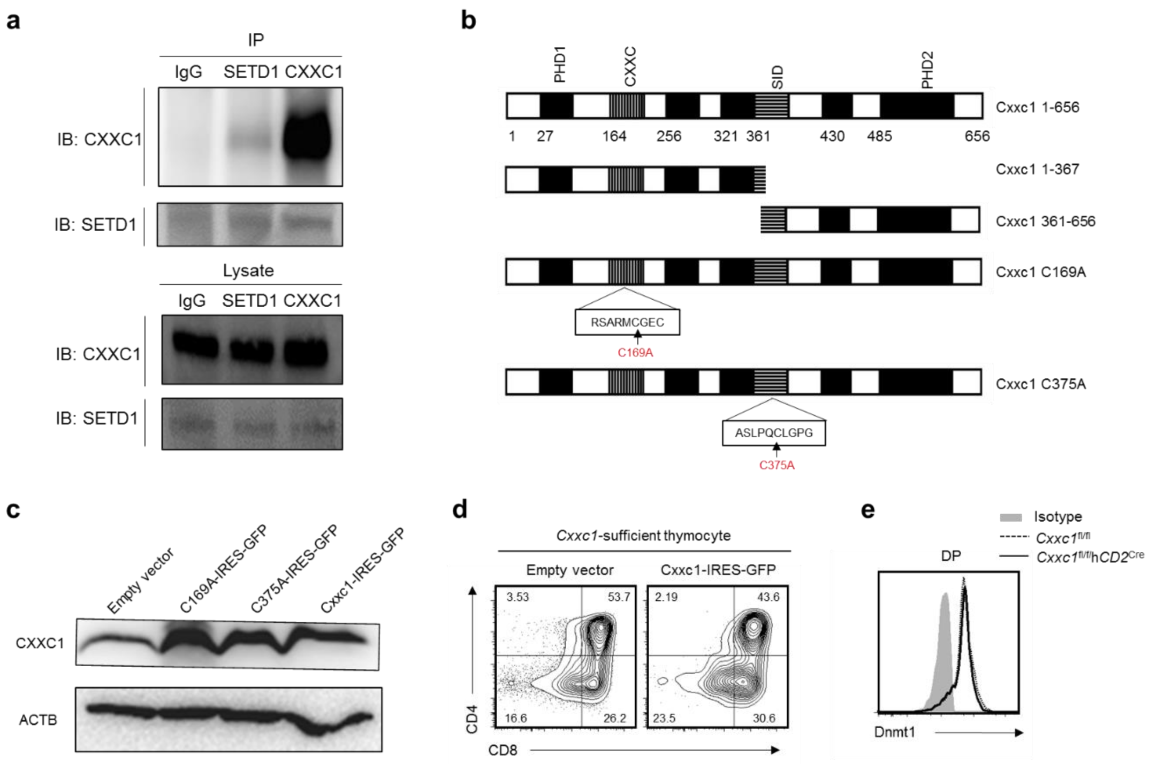

Supplementary Figure 4

The Setd1 interacting domain of Cxxc1 is required for intrathymic T cell development

(a) DP thymocytes were sorted from wild-type mice, then lysed and immunoprecipitated with anti-Cxxc1, anti-Setd1 or rabbit immunoglobulin G (IgG; control antibody) and detected with anti-Cxxc1 or anti-Setd1 (top), immunoblot analysis of lysates (without immunoprecipitation) with anti-Cxxc1 or anti-Setd1 (bottom). Data is from three independently sorted DP cell (each from 1 mouse). (b) Schematic view of CXXC1 protein and strategies of generation of mutated and truncated CXXC1 proteins. (c) Wild-type DN3 cells were transfected with indicated retrovirus and were co-cultured with OP9-DL1 cell line; GFP positive cell were sorted after 5 days and subjected to Western Blot analysis. Data comes from two separately sorted DN3 cell (each from 2 mice). (d) DN3 cells from wild-type mice were sorted and transfected with indicated retrovirus, GFP^+ thymocytes were analyzed by FACS after co-culture with OP9-DL1 for 5 days. Data comes from two separately sorted DN3 cell (each from 1 mouse). (e) Detection of DNMT1 protein expression in DP thymocytes. Thymocytes from WT or *Cxxc1*-deficient mice were permeabilized with Foxp3 staining buffer, followed by staining with anti-DNMT1 primary antibody and anti-mouse IgG fluorescein–conjugated secondary antibody. Data is from independent three mice experiments

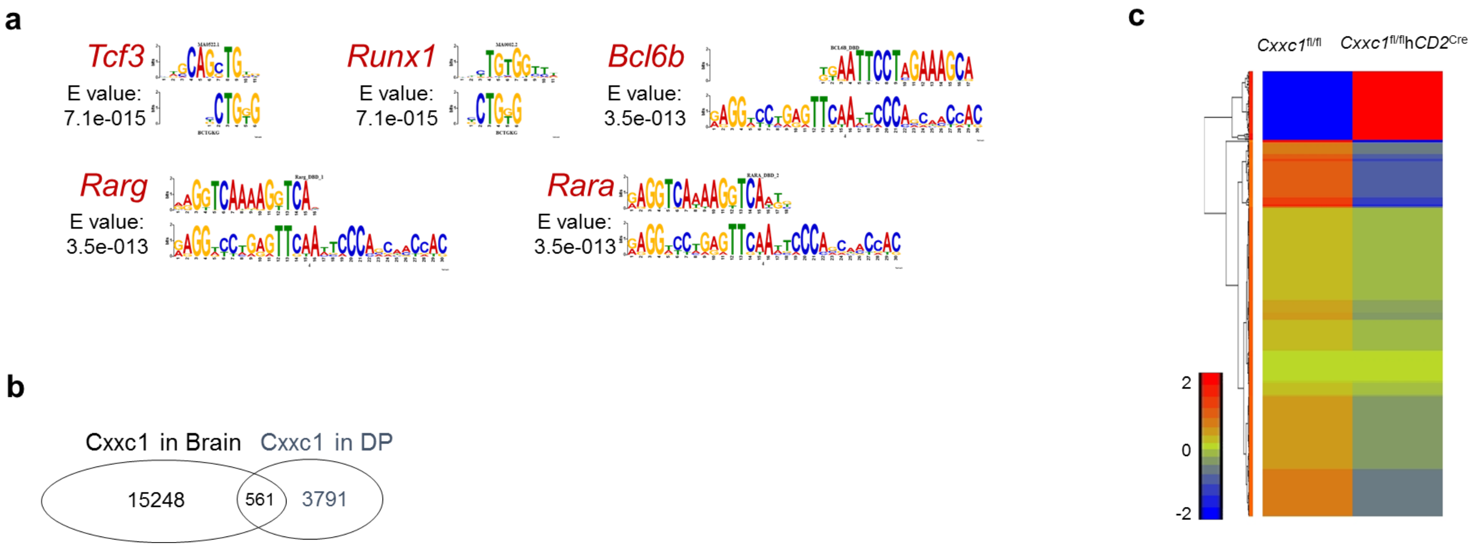

Supplementary Figure 5

Cxxc1 binding sites and Cxxc1 mediated gene expression regulation

(a) Enriched binding motifs among Cxxc1 binding sites in DP thymocytes.
(b) Overlap between Cxxc1 binding sites in DP thymocytes and brain tissues.
(c) Hierarchical clustering of 387 Cxxc1 target genes with at least 2 fold expression difference between WT and *Cxxc1*-deficient DP thymocytes.

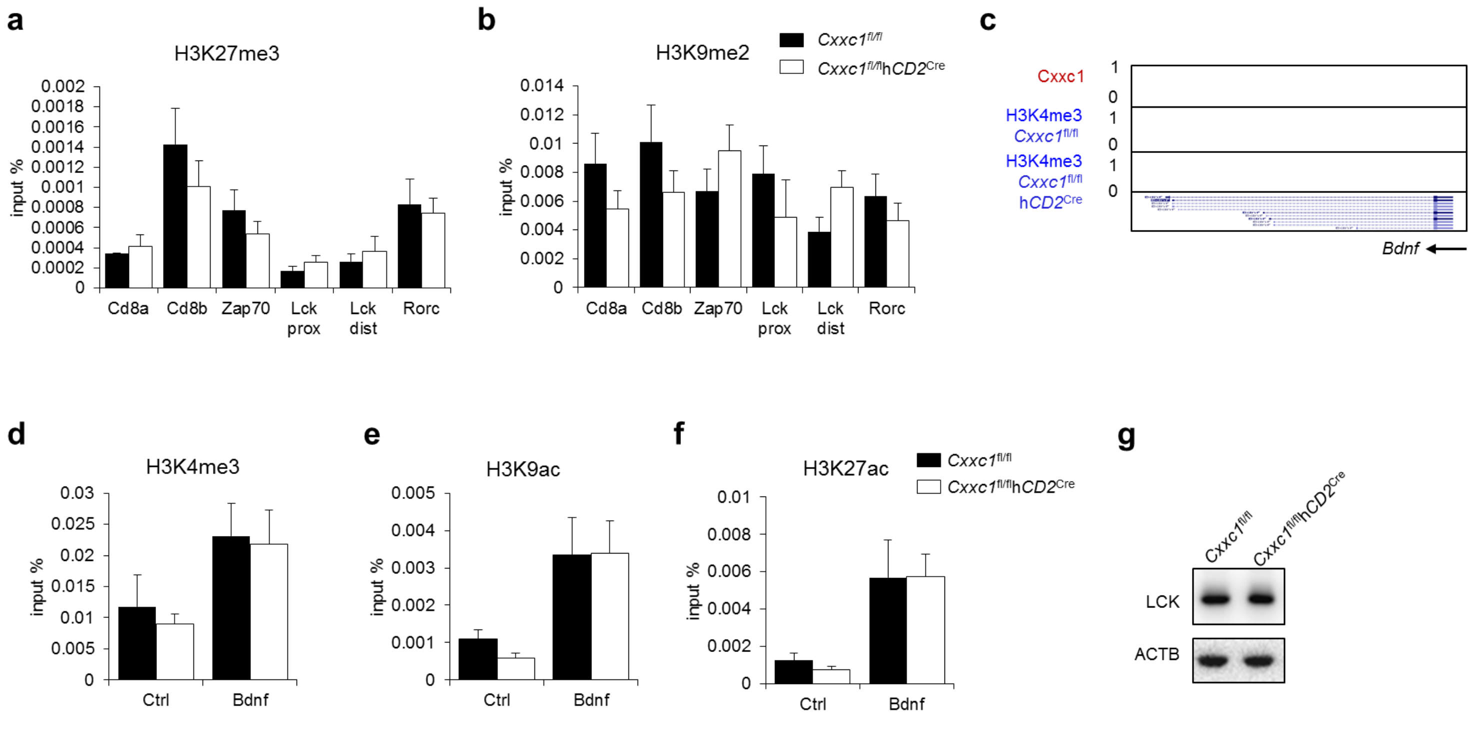

Supplementary Figure 6

Epigenetic regulation of Cxxc1 target loci

(a, b) H3K27me3 (a) and H3K9me2 (b) modifications on *Cd8a-Cd8b*, *Zap70*, *Lck* and *Rorc* loci in wild-type and *Cxxc1*-deficient DP thymocytes, detected by ChIP-PCR assays. Data is from three independently sorted DP cell sets (each from 2 mice). (c) Cxxc1 binding profile and H3K4me3 on the locus of *Bdnf* gene in wild-type and *Cxxc1*-deficient DP thymocytes. (d-f) H3K4me3 (d), H3K9ac (e), and H3K27ac (f) modifications on *Bdnf* locus in wild-type and *Cxxc1*-deficient DP thymocytes. Experiments are same as Fig. 4 (d-f). Error bars indicate s.d. (g) Detection of Lck protein level in indicated DP thymocytes by Western Blot. Data comes from three independently sorted DP cell sets (each from 2 mice).

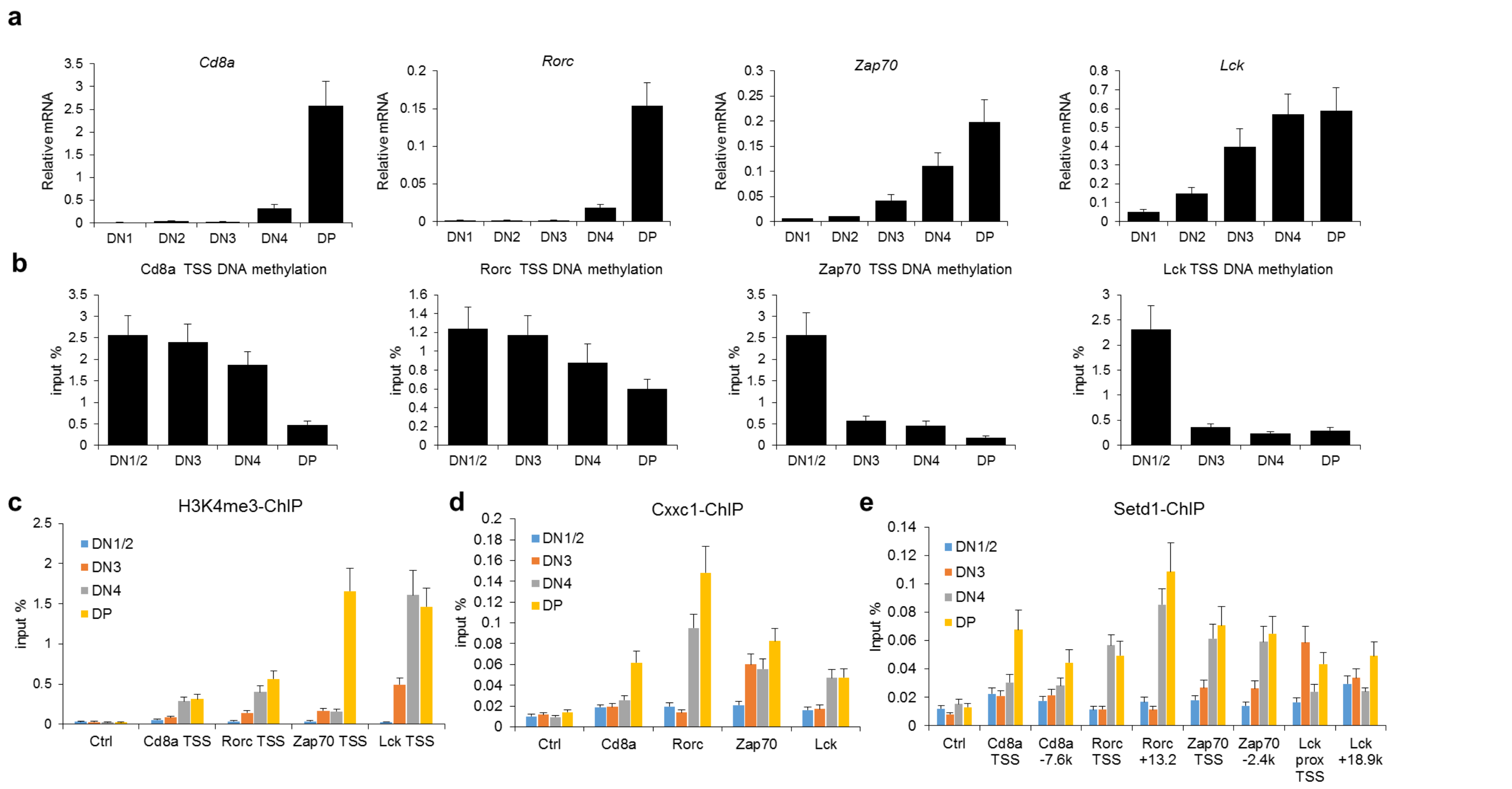

Supplementary Figure 7

Profile of expression, DNA methylation and binding of Cfp1, Setd1, H3K4me3 during T cell development.

(a) Indicated populations sorted from wild-type mice were subjected to Real-time Quantitative PCR analysis. Data is from independently sorted samples (each from 2 mice). (b) Detection of DNA methylation on promoter regions of indicated genes from different populations of wild-type mice were analyzed by MeDIP assays. DN1/2: DN1 mixed with DN2 cell. Data comes from independently sorted samples (each from 3 mice). (c-e) Analysis of H3K4me3 modification(c), binding of Cxxc1, (d) and Setd1 (e) by ChIP-qPCR on *Cd8a, Rorc, Zap70* and *Lck* loci in indicated populations. DN1/2: DN1 mixed with DN2 cell. Data comes from three independently sorted samples (for H3K4me3, each from 2 mice; for Setd1 and Cxxc1, each from 4 mice). Errors bars indicate s.d.

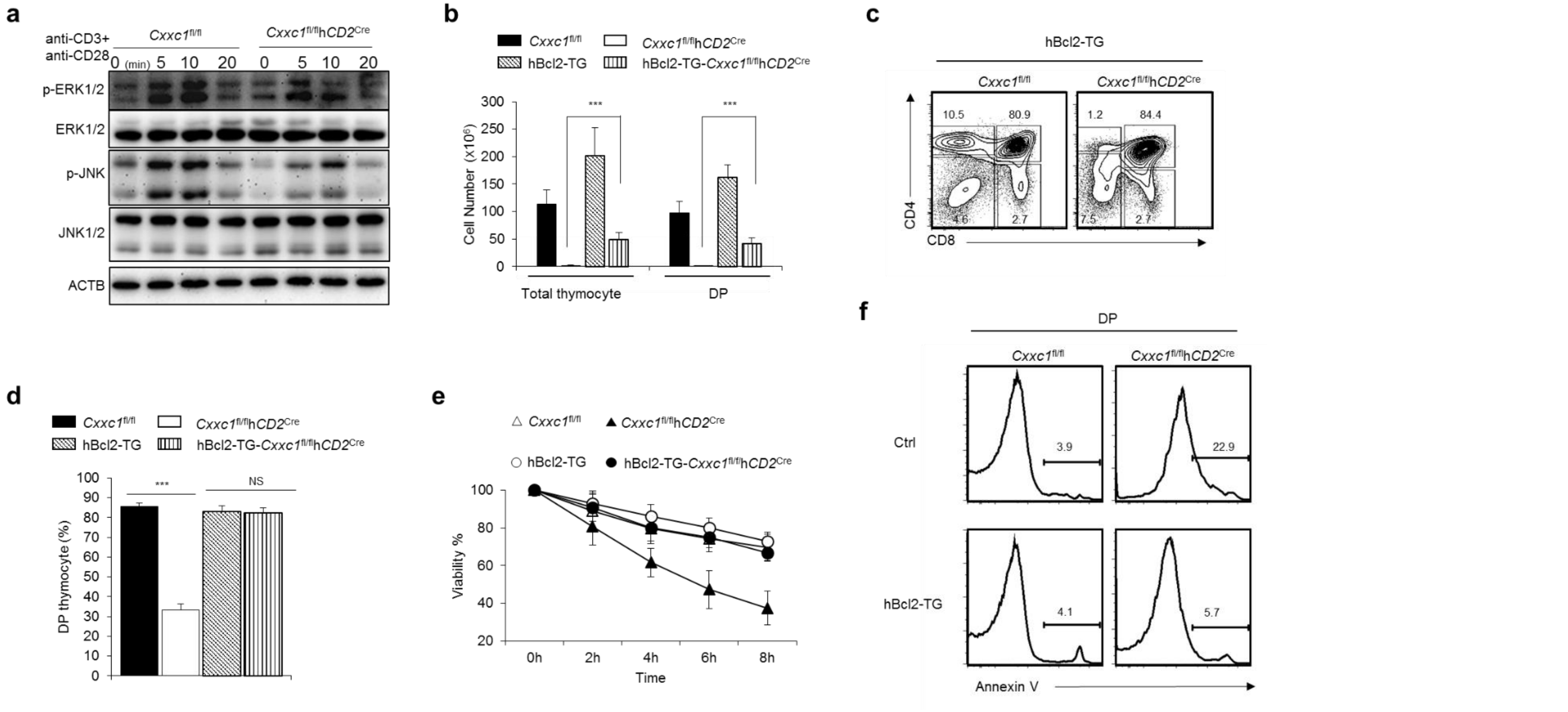

Supplementary Figure 8

Bcl2 transgene could restore the survival of *Cxxc1*-deficient DP thymocytes but not TCR signaling

(a) DP thymocytes sorted from wild-type or *Cxxc1*-deficient mice were stimulated with anti-CD3 plus anti-CD28 antibodies for indicated time. The cell lysate were subjected to Western Blot using indicated antibodies. Data is from three independently sorted DP cell sets (each from 5 mice). (b) Quantitation of thymocyte subpopulations in indicated mice. The statistical significance were determined by unpaired t-test (two-tailed). ****P* <0.001. (c) Flow cytometry analysis of thymocytes from wild-type or *Cxxc1*-deficient mice on hBcl2 transgenic background. (d) Percentage of DP thymocytes in indicated mice was shown. The P value were calculated by unpaired t-test (two-ailed). NS, not significant. ****P*<0.001. Data comes from six mice analysis (b-d). (e) DP thymocytes sorted from wild-type, *Cxxc1*-deficient, hBcl2-TG or hBcl2-TG-Cxxc1-deficeint mice were cultured and harvested at indicated time. Viability was measured using flow cytometry by staining of Annexin V. Data comes from three independently sorted DP cell sets (each from 3 mice). Results are presented as mean ± s.d. (f) Annexin V staining of thymocytes from indicated mice following staining of anti-CD4 and anti-CD8 antibodies, see (c).

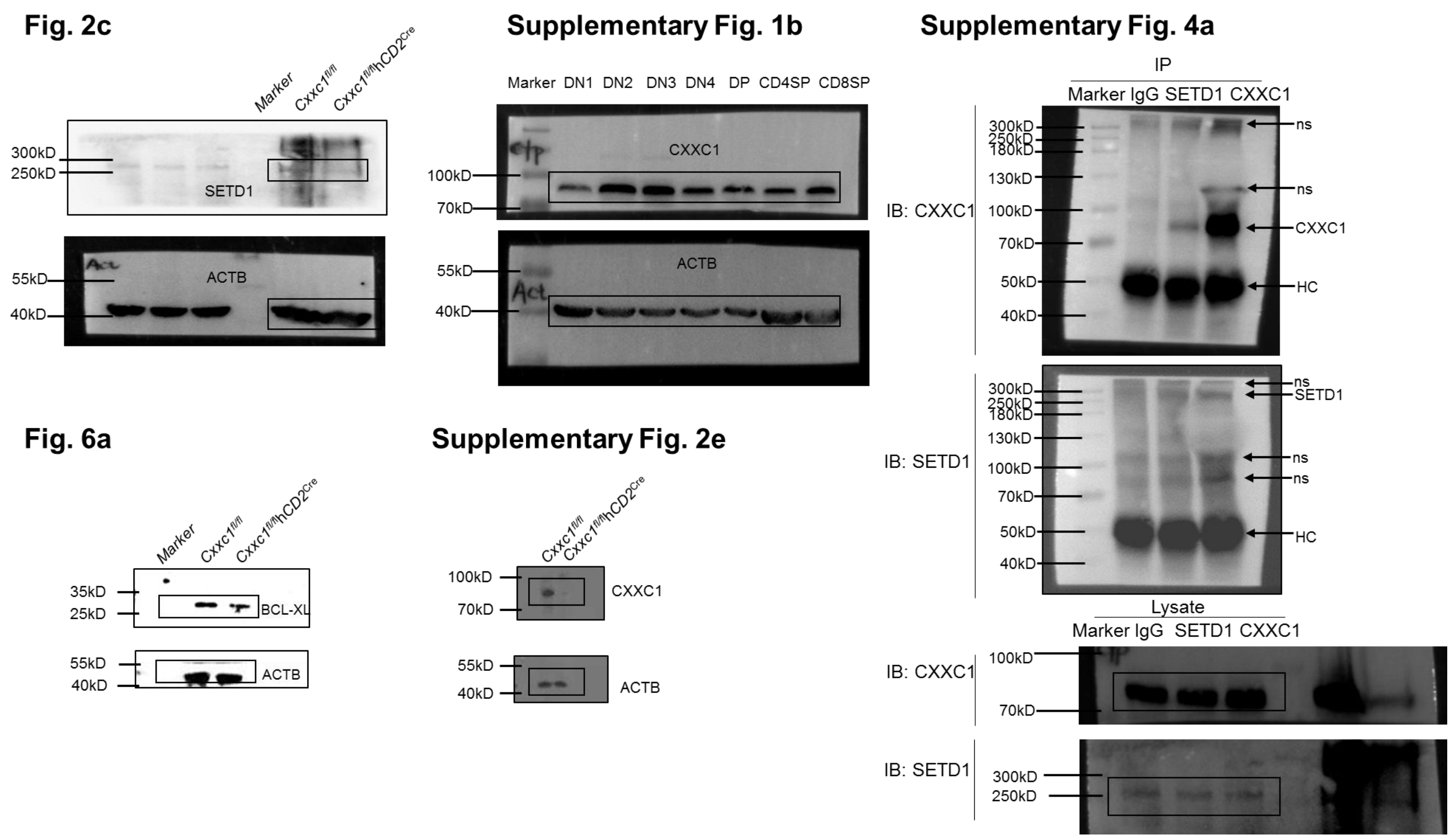

Supplementary Figure 9

Full-size Western Blots.

Supplementary Fig. 4c

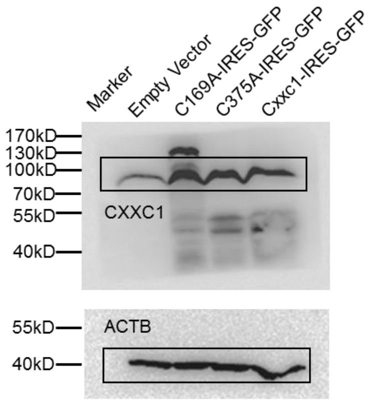

Supplementary Fig. 6g

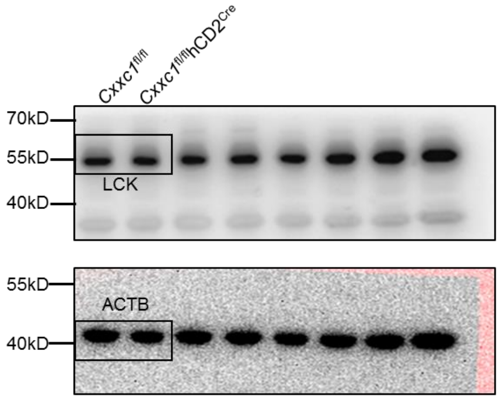

Supplementary Fig. 8a

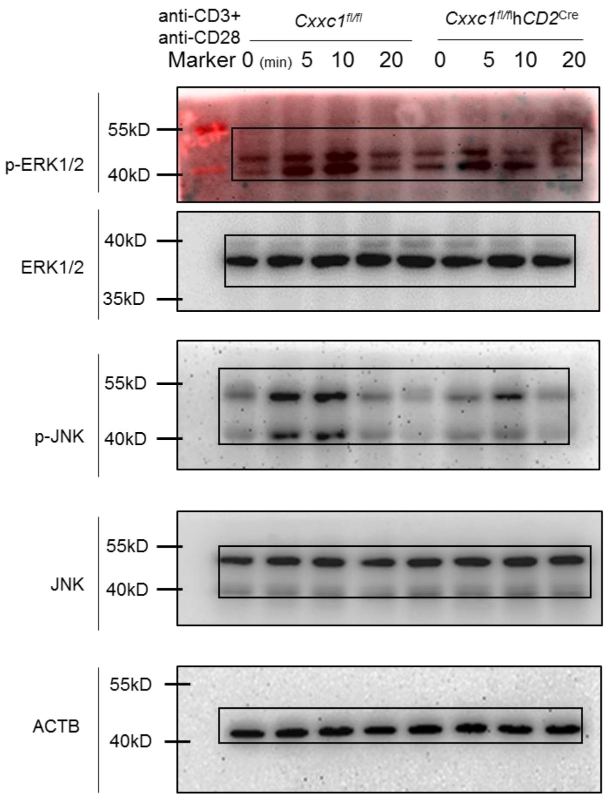

Supplementary Figure 9 (continued)

Full-size Western Blots.

Supplementary Tables

Table S1: Summary of ChIP-seq experiments

	Total unique tag count	Total number of peaks	Promoter peak	Exon peak	Intron peak	Intergenic peak
H3K4me3 *Cxxc1*$^{fl/fl}$	9,460,792	14,237	2244 (16%)	2414 (17%)	5038 (35%)	4494 (32%)
H3K4me3 *Cxxc1*$^{fl/fl}$h*CD2*^{Cre}	9,448,321	2,979	466 (16%)	316 (11%)	1081 (36%)	1106 (37%)
Cxxc1 ChIP *Cxxc1*$^{fl/fl}$	9,106,310	4,352	208 (5%)	177 (4%)	1486 (34%)	2470 (57%)
Cxxc1 ChIP *Cxxc1*$^{fl/fl}$h*CD2*^{Cre}	5,913,588	-	-	-	-	-
Input *Cxxc1*$^{fl/fl}$	2,400,743	-	-	-	-	-

Table S2: Gene Ontology analysis of 387 Cxxc1 target genes

GO Term	Gene Count	P Value	Genes
Chromatin modification	18	5.2E-06	SATB1, DNMT3A, TBL1XR1, CTCF, SUZ12, KDM1A, HDAC2, HDAC1, BRE, KDM3A, ACTL6A, TLK2, KDM3B, RNF20, HDAC7, KDM4D, SUDS3, SUV420H1
T cell differentiation	10	2.0E-05	EGR1, CARD11, PTPRC, SATB1, CD8A, BCL11B, LCK, ZAP70, RORC, CD74
T cell activation	11	1.1E-04	EGR1, CARD11, PTPRC, SATB1, CCND3, CD8A, BCL11B, LCK, ZAP70, RORC, CD74
Leukocyte activation	14	4.6E-04	EGR1, PTPRC, SATB1, CD8A, RORC, PRKCD, CD74, CARD11, CCND3, BCL11B, LCK, ZAP70, HDAC7, LCP2
Thymic T cell selection	4	3.0E-03	CARD11, PTPRC, ZAP70, CD74
Covalent chromatin modification	8	3.1E-03	SUZ12, KDM1A, DNMT3A, SATB1, HDAC2, HDAC1, ACTL6A, SUV420H1
T cell differentiation in the thymus	5	3.6E-03	CARD11, PTPRC, ZAP70, RORC, CD74

Table S3: Primers for detecting the homologous recombination arm, loxp site and deleted band in Cxxc1 locus:

**P1**	GTGGAAGTTTAAAGTCATCCTGGGC
**P2**	CTGAGCCCAGAAAGCGAAGGA
**P3**	CCTCCCCCGTGCCTTCCTTGAC
**P4**	CTCCAACTAGACTTCTACTCCTCCAGC
**P5**	CGAGAGATGAAGAGGAGCCA
**P6**	CTGAATGGTCCTAGAACCTC
**P7**	AGCTTGCGGAACCCTTCGAA
**P8**	CACAAAGATAGGCTCCATCC

Table S4: Primers for ChIP:

Locus	Location	Forward Primer	Reverse Primer
**Ctrl**	Bdnf -9.4K	GGAACATCTCCACAGTAATTTGC	CCTAGAGGTTAAACAACTTCAGC
**Zap70**	TSS	CTCATTCAGTGTTCAGGGAC	GTATAGGGTGGTTCAGATGC
	-2.4K	GCTTGGCAGCTTTCCGTACC	TAGCCAGACATTGTGGGATTAGC
**Rorc**	TSS	ACCTGTGTGGAGCAGAGCTT	TGCCCCCATTCACTTACTTC
	+13.2K	ATGGTTTCCCTTTCTCCACA	CAGGGAGTGGCTCCAGATAG
**Lck**	prox TSS	AAAGTACCCGAGATCGTCAG	AGAGGGAACCCAGTCAGGAG
	dis TSS	CAGATGGCAGAGGCAAGAGC	GTGGGAACCAGACAGCAGAA
	-18.9K	GAGTCTCAGTTGCCCAGAGG	GGTGTAGCTCGGTGGAAGAG
**CD8a**	TSS	TTCATCCCACAACAAGATAACG	TTCTGAAGGACTGGCACGAC
	-7.6K	TCCAGATATGCCCAAAGTCC	GATCAGTGAGCCAGCTTTCC
**CD8b**	TSS	GACAGAGCACTGAGGGGAAC	TAGTGCAGATTGGGGGTAGC
**Bdnf**	TSS	CCTTTCTATCATCCCTCCCG	GGCTCTTCGATCTAGAAAGGAC

Table S5: Primers for qPCR:

Gene	Forward Primer	Reverse Primer
**GAPDH**	ACTCCACTCACGGCAAATTCA	GCCTCACCCCATTTGATGTT
**Cxxc1**	ATCCGGGAATGGTACTGTCG	CTGTGGAGAAGATTTGTGGG
**Bcl-xl**	GTCGCCGGAGATAGATTTGAAT	GTCGCCGGAGATAGATTTGAAT
**CD8a**	CCGTTGACCCGCTTTCTGT	CGGCGTCCATTTTCTTTGGAA
**CD8b**	CTCTGGCTGGTCTTCAGTATGA	TCTTTGCCGTATGGTTGGTTT
**Zap70**	CTACGTGCTGTCGTTGGTG	GTTACACGGCTTACGCAGGT
**Lck**	AAGGTGGCGGTGAAGAGT	TTCCGTGATGATGTAGATGG
**Rorc**	CCCCAGTGACACCATTCT	TTAGGGACAGAGGACAGC

Table S6: Primers for MeDIP:

Gene	Location	Forward Primer	Reverse Primer
**Cd8a**	+1	GCTGGCTAAAGGAGCAGTTT	AGCAAGCAGCTTAGGTCAAG
**Rorc**	-161	TACTCAGGAGAGGGGATTTC	TCCAAAACCACAGCTACAGC
**Zap70**	+202	CAAGTGGTAGGGACCTGAGA	CTGCCTGTGCTGCTTTCTAA
**Lck**	+12	AGTCAGGAGCTTGAATCCCA	ACATCAGACATCCTAGAGCC
